# Electrostatic charge at the biomaterial-pathogen interface influences antibiotic efficacy

**DOI:** 10.1007/s44307-025-00061-z

**Published:** 2025-04-03

**Authors:** Andrew Hayles, Huu Ngoc Nguyen, Markos Alemie, Jitraporn Vongsvivut, Neethu Ninan, Richard Bright, Panthihage Ruvini Dabare, Christopher Gibson, Vi Khanh Truong, Krasimir Vasilev

**Affiliations:** 1 Biomedical Nanoengineering Laboratory, Flinders University, Bedford Park, SA 5042 Australia; 2 School of Biomedical Engineering, Faculty of Engineering, University of Sydney, Sydney, NSW 2050 Australia; 3 Infrared Microspectroscopy (IRM) Beamline, ANSTO ‒ Australian Synchrotron, 800 Blackburn Road, Clayton, VIC 3168 Australia; 4 Academic Unit of STEM, University of South Australia, Mawson Lakes, SA 5095 Australia; 5 Flinders Microscopy and Microanalysis, Flinders University, Bedford Park, SA 5042 Australia; 6 Adelaide Microscopy, the University of Adelaide, Adelaide, SA 5000 Australia; 7 Deaprtment of Biomedical Engineering, Healthcare Engineering Innovation Centre, Khalifa University, Abu Dhabi, United Arab Emirates

**Keywords:** Antibiotic prophylaxis, Surface charge, Drug tolerance, Biomaterials, Coating, Nanotechnology

## Abstract

Implant-associated infections (IAI) are a considerable burden for healthcare systems globally. While novel anti-infective biomaterials are being pursued, prophylactic antibiotic treatment remains the most important intervention for mitigating IAI. The antibiotic tolerance of bacteria has been widely studied, but until recently, the contributions of biomaterial-pathogen interactions have been overlooked. In the present study, we investigate how material electrostatic charge influences the physiological state of the most clinically challenging pathogen—*Staphylococcus aureus*, and the implications on its antibiotic tolerance. We utilized a combination of techniques, including quantitative gene expression and synchrotron-sourced attenuated total reflectance Fourier-transform microspectroscopy, to characterize this phenomenon – elucidating how surface attachment to differently charged substrates drives the pathogen to modify its phenotype. Subsequently, we found a direct relationship between the activity of oppositely charged antibiotics (vancomycin and cefazolin) and the biomaterial-pathogen interface, which we determined to be governed by material electrostatic properties. The findings of the present study have the potential to inform the development of enhanced procedures of antibiotic prophylaxis by instructing personalized biomaterial-antibiotic pairing strategies. These new insights hold promise to contribute to reducing the rate of IAI by enabling clinicians and surgeons to maximize the efficacy of prophylactic antibiotic treatments during implant placement procedures.

**Supplementary Information:**

The online version contains supplementary material available at 10.1007/s44307-025-00061-z.

## Introduction

Despite decades of advancements in medicine, technology and surgical practices, the burden of implant-associated infections remains a considerable and unrelenting threat. The outcomes of such infections include chronic pain, delayed healing, implant rejection, amputation, sepsis and death (Trampuz and Widmer 2006). Microbial contamination of implantable devices occurs via multiple pathways, one of the primary routes being the direct transfer of pathogens from the skin surrounding the surgical site during implant placement (Schierholz and Beuth 2001). A significant focus has been on developing anti-infective technologies and techniques to address this issue. These include improved sterilization protocols and various strategies to confer antibacterial properties to biomedical materials, such as coating with antifouling polymers (Cavallaro et al. 2016), silver nanoparticles (Taheri et al. 2014; Vasilev et al. 2010b), and topographical modifications (Bright et al. 2023, Hayles et al. 2022). Some of these have proven to be highly potent. Unfortunately, despite the reported efficacy, many emerging biotechnology and biomaterial developments are limited by regulatory considerations and challenges in manufacturing. While these issues are being resolved, prophylactic antibiotic treatments remain the most important and impactful intervention for mitigating infection. It is a standard practice to administer an oral or intravenous dose of antibiotics before implant placement surgery (Parvizi et al. 2013). The most common drug used in surgical prophylaxis is cefazolin, a cell wall active ß-lactam inhibiting the peptidoglycan crosslinking enzyme penicillin-binding protein (PBP). However, the most common alternative is vancomycin when the patient is a known host of multidrug-resistant *Staphylococcus aureus* or has an allergy to ß-lactams (Crawford et al. 2012). Vancomycin is a cell wall-active glycopeptide that binds to the D-ala-D-ala residues of peptidoglycan, preventing crosslinking, ultimately leading to lysis of the pathogen.

Recent research from our group has revealed the existence of a relationship between material surface properties and an underappreciated form of antibiotic tolerance (Hayles et al. 2024). Specifically, this form of antibiotic tolerance involves antibiotics bearing an electrostatic charge, such as vancomycin and cefazolin. It has long been established that *S. aureus,* among other Gram-positive bacteria, possesses biological systems capable of modifying the electrostatic properties of the cell surface. This is achieved through multiple routes. In one route, the products encoded by the *dlt* operon enable *S. aureus* to attach positively charged D-alanine residues to the teichoic acids protruding outwardly from the cell. As teichoic acids bear a net negative charge, the addition of the positively charged D-alanine dampens the negativity of the cell surface (Koprivnjak et al. 2006). Similarly, the membrane-bound protein MprF enables the biosynthesis of lysyl-phosphatidylglycerol (LPG), which is a positively charged phospholipid that is incorporated into the lipid bilayer of the cytoplasmic membrane (Ernst et al. 2009). Similar to the actions of the *dlt* operon, the insertion of LPG into the cell membrane shifts the overall cell surface charge in the positive direction. Both processes have previously been implicated in tolerance to the positively charged antibiotic daptomycin (Bayer et al. 2013). This is intuitive because a reduction in the net negative cell surface charge would be expected to reduce the attractive potential of the cell surface toward positively charged compounds such as daptomycin. In our previous research, we observed that the expression of *mprF* and the *dlt* operon are influenced by surface-attachment (Hayles et al. 2024). This is most likely a central process of biofilm formation. In support of this interpretation, other studies have investigated the role of electrostatics in biofilm stabilization. For example, Graf and colleagues (Graf et al. 2019) showed that *S. aureus* utilizes positively charged secretions to interface with anionic cell surface components – thereby acting as an electrostatic bridge to reinforce the structural integrity of the biofilm. It is becoming increasingly clear that electrostatic dynamics within the biofilm structure also have important implications for antimicrobial treatment, as the upregulation of *mprF* and the *dlt* operon enhanced the tolerance of *S. aureus* to the positively charged vancomycin (Hayles et al. 2024). Interestingly, while the upregulation of *mprF* and *dlt* on adherent *S. aureus* causes enhanced tolerance to vancomycin, the same effect was not observed for cefazolin. Cefazolin operates via a mechanism similar to vancomycin but bears a negative charge at physiological pH. Thus, it was determined that the difference in tolerance to these drugs was attributable to the electrostatic interactions between the cells and the antibiotics.

Inspired by the knowledge that *S. aureus* actively modulates its cell surface charge, we hypothesized that the pathogen would adapt to material surfaces with differing electrostatic properties to maximize biofilm formation. We also hypothesize that the downstream effect would be that the activity of different antibiotics would be, at least in part, influenced by the electrostatic properties of the material to which the pathogen has adhered. We investigated this using plasma-polymer deposition to fabricate material surface coatings with varying electrostatic charges. We then analyzed the response of *S. aureus* and the sensitivity of the pathogen to antibiotics upon attachment to these surfaces. We measured the expression of genes related to cell surface charge and synchrotron-sourced attenuated total reflectance (ATR) Fourier-transform infrared (FTIR) microspectroscopy to characterize the changes to the cell surface in response to material surface charge. We then evaluated the performance of oppositely charged antibiotics (cefazolin and vancomycin). We interpreted these results in the context of the observed interactions between the material surfaces and bacterial cell characteristics.

## Results and discussion

### Fabrication and characterization of charged substrates

**Fig. 1:**
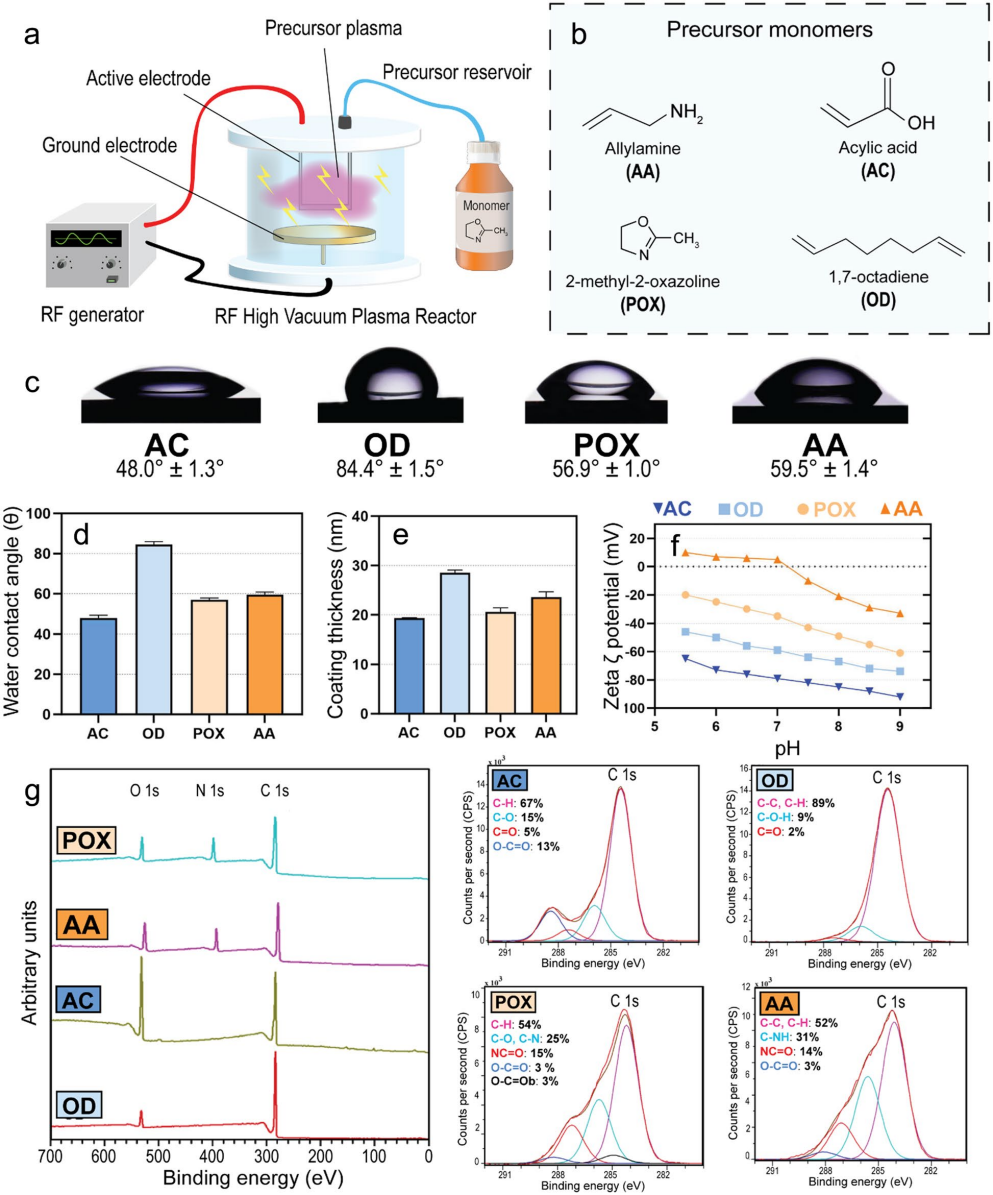
Fabrication and characterization of plasma-polymer coated glass coverslips. **a** Schematic of the custom-built RF low-pressure plasma system used for coatings deposition. **b** Molecular structure of the precursors used for plasma polymer deposition. **c** Photographs showing water droplets spreading on the plasma-polymer-coated glass coverslips. **d** Water contact angle measured using sessile water droplet goniometry. **e** Thickness of the plasma-polymer coatings measured by ellipsometry. **f** Streaming ζ-potential measurements. **g** XPS analysis of the four coatings – survey and C1s high-resolution spectra

To study the influence of material surface charge on the efficacy of antibiotics against *S. aureus*, we deposited thin film coatings on model substrates by plasma-polymerization utilizing four different precursors, namely, 1,7-octadiene (OD), 2-methyl-2-oxazoline (POX), acrylic acid (AC) and allylamine (AA) (Fig. 1). These four precursors were chosen because their deposition products are known to exhibit a range of different electrostatic charges. We first performed extensive physical, chemical and topographical characterization of the modified surfaces. Hydrophilicity was measured by sessile drop static water contact angle (Fig. 1 c and d). The samples coated using 1,7-octadiene (OD) as a precursor for deposition displayed the highest water contact angle of 85° ± 1°, indicating the greatest hydrophobicity amongst the four coatings in the set. The water contact angle of samples coated using allylamine (AA) was 60° ± 1°, 2-methyl-2-oxazoline (POX) was 56° ± 1.0°, and acrylic acid (AC) was 48.0° ± 1.3°. The coating thickness was measured by ellipsometry (Fig. 1e). All coatings were in the 20–30 nm thickness range, with negligible variance between replicates (± 1 nm). This was important to ensure that the chemistry of the underlying substrate played no role in the subsequent biological measurements. To determine the overall charge of the modified surfaces, we measured their zeta (ζ)-potential (Fig. 1f). For all plasma-coated samples, increasing the pH from 5.5 to 9 shifted the ζ-potential in the negative direction. At pH 7, the ζ-potential measurements were + 5 mV for AA, −35 mV for POX, −59 mV for OD and −79 mV for AC. The results are consistent with published studies (Mierczynska et al. 2012; Visalakshan et al. 2019; Mierczynska-Vasilev et al. 2019). The AA coating contains a population of amine groups that protonate to deliver a positive surface charge (Vasilev et al. 2010a; Mierczynska et al. 2012). AC is rich in carboxylic acid, which deprotonates and results in a negative surface charge (Vasilev et al. 2010a; Mierczynska et al. 2012). The negative surface charge on the OD and POX surface can be attributed to oxidation during plasma deposition and exposure to ambient air (Fig. S1 and Table S1). To characterize the chemical composition of these polymer films, we used X-ray photoelectron spectroscopy (XPS) (Fig. 1g). All four plasma-modified surfaces exhibited peaks attributable to O1s and C1s, while the POX and AA surfaces showed an additional peak attributable to N1s. The atomic concentration of these elements was consistent with published studies (Ruvini L Dabare et al. 2022). To further elucidate the chemical composition of the modified surfaces, we analyzed high-resolution spectra in the C1s region, which was deconvoluted using the following components. All four coatings exhibited a characteristic peak assigned to aliphatic hydrocarbons (C–C, C-H) at 285 eV. The highest hydrocarbon proportion (89 At%) was detected on the OD coating, which is expected based on the chemical composition of the precursor. The high-resolution C1s spectrum of the AC coating had a clear component at 288.5 eV, which can be assigned to O-C = O bonds, suggesting that the carboxyl functional group was present in the plasma-polymer as intended. The component at 287.2 eV was assigned to N–C = O (amide functional groups) on the POX surface, which accounted for 15% of the carbon bonds identified in the high-resolution C 1S spectra, consistent with published reports (Ramiasa et al. 2015). Finally, on the AA-modified surface, we observed a strong presence of C-N bonds (286 eV), which can be attributed to amine functional groups. The XPS data also confirmed the absence of contaminants, which is vital for the forthcoming biological experiments. The moderate presence of oxygen in the AA and OD coatings was attributed to the exposure of the modified surfaces to ambient air during and after plasma deposition, consistent with published literature (Gallino et al. 2010). The lack of silicon signal in the XPS spectra further confirms that the thickness of the coating was greater than the sampling depth of XPS (ca 10 nm) and that the coatings are continuous and pinhole-free. To support the XPS characterization of the plasma-polymer films, we also used Fourier-transform infrared (FTIR) spectroscopy to characterize the chemical bonds in the plasma-polymer coatings (Fig. S2). The FTIR spectra corroborated the XPS measurements. Specifically, FTIR spectra of AC detected the signature of the carboxyl group in the band around the 1572 cm^−1^ region (attributed to C = O stretching vibration) (Alavi et al. 2011) and a broad peak spanning the region from 3675 to 2646 cm^−1^ (attributed to O–H stretch) bonds, and a C-O bond at 1130 cm^−1^.(2002) The OD coating exhibited bands consistent with the hydrocarbon nature of the precursor, including a band at 1380 cm^−1^ (C = C bond), 2960 cm^−1^ (C-H stretching) and 3100–3000 cm^−1^ (C-H stretching) (Siow et al. 2017). The POX coating showed bands at 1750 cm^−1^ and 1350 cm^−1^, characteristic of CH_3_ and CH_2_ groups, as well as C = N which is indicative of the oxazoline ring (Macgregor and Vasilev 2019). Lastly, the AA coating displayed a broad band at 3500–3000 cm^−1^ (attributed to N–H stretching in primary and secondary amine groups).

Prior to moving on to the biological analyses, it was also necessary to analyze the topography of the plasma-polymer-coated substrates. This was important to ensure that all biological measurements were influenced only by surface chemistry and not by topography, as we have previously shown a relationship between surface topography and the expression of genes associated with the surface charge of *S. aureus *(Hayles et al. 2023). Table 1 shows roughness measurements acquired by atomic force microscopy (AFM) across 10 × 10 µm scans and confirms that the coatings did not have significant variations. AFM micrographs of the four different coatings are presented in Fig. S3.

**Table 1:** Roughness measurements of plasma-modified glass coverslips

Plasma-polymer	Ra (average roughness, nm)	Rq, RMS (root mean square, nm)
AC	0.52 ± 0.66	0.23 ± 0.03
OD	0.47 ± 0.03	0.31 ± 0.02
AA	0.94 ± 0.04	0.52 ± 0.01
POX	0.78 ± 1.32	0.24 ± 0.07

Lastly, although these four surface coatings are intended to stand as model surfaces representing a range of surface electrostatic properties, we verified their in vitro cytocompatibility with THP-1 monocyte-like cells (Fig. S4) For AC, OD, POX and AA, the THP-1 cells retained a viability of approx. 93%, 86%, 99% and 93%, respectively, after 72 h incubation. Under the conditions tested, these data indicate a high degree of biocompatibility, and therefore support their potential use in clinical implant applications.

### Initial attachment of *S. aureus* to differently charged substrates

**Fig. 2:**
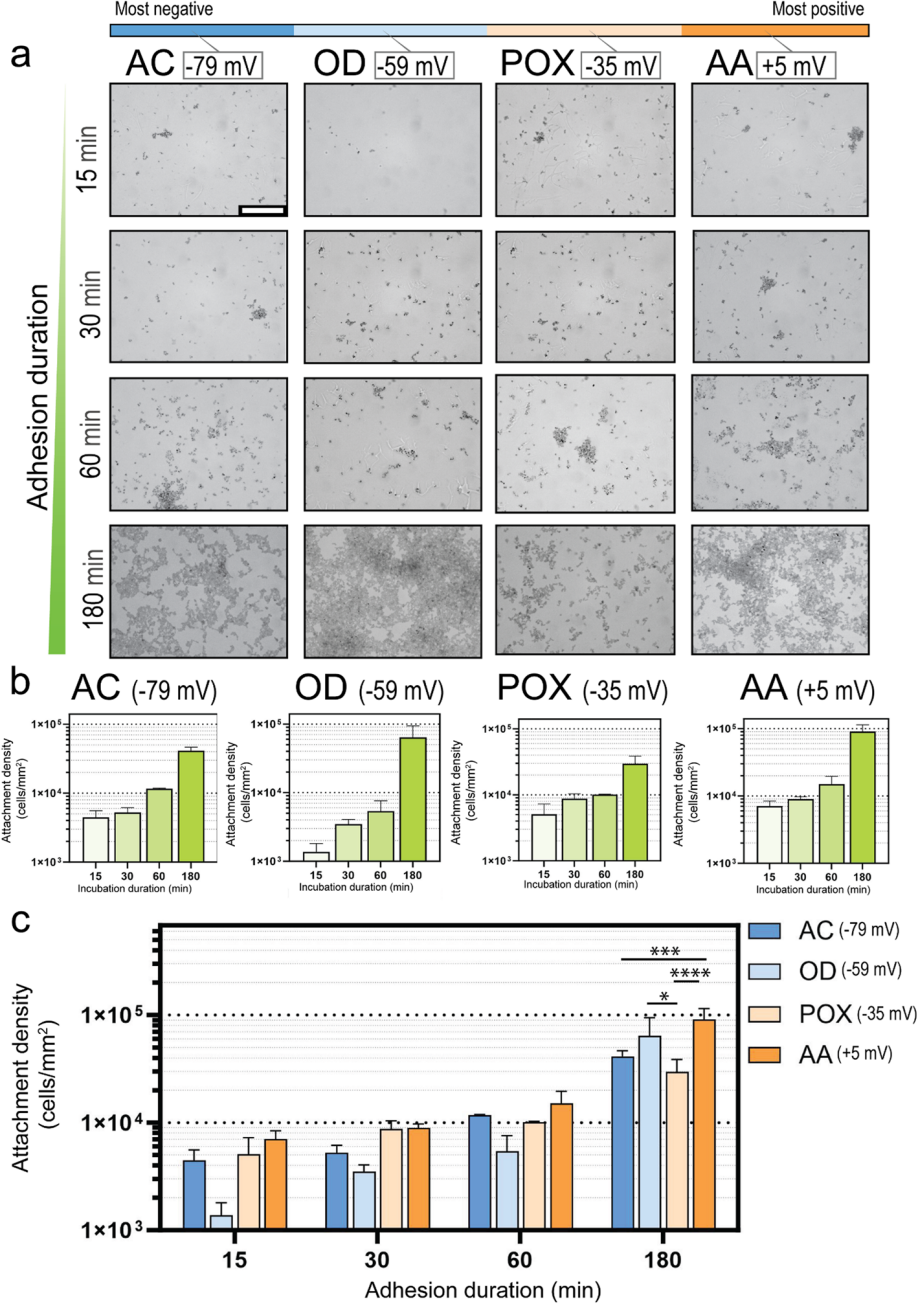
Rate of surface establishment of *S. aureus* on plasma-modified substrates with a range of electrostatic charges. **a** Light micrographs (100 × magnification) of *S. aureus* on the plasma-polymer coated slides. The scale bar represents 50 µm. **b** Attachment density measurements (cells/mm.^2^) as a function of time for each material coating. **c** Comparison of cell density between the different plasma-coated glass slides. Data plotted as mean ± SD, *n* = 3 * *P* < 0.05, *** *P* < 0.001 and **** *P* < 0.0001

Next, we investigated the influence of substrate surface charge on the attachment rate of *S. aureus* between 15 and 180 min during the early stages of adhesion (Fig. 2). We chose to study surface attachment during this early period for two reasons. Firstly, electrostatic interactions are most relevant to the very earliest stages of surface attachment (Moormeier and Bayles 2017). Secondly, the initiation period of surface attachment is when prophylactic antibiotics are expected to play the most significant role. To characterize the attachment behavior of *S. aureus* on our model surfaces, we incubated the plasma-polymer-coated glass coverslips in a suspension of *S. aureus* for different incubation periods. Then, we analyzed the density of adherent cells under a light microscope at 100 × magnification. As a general observation, all samples gradually accumulated cells in the first 60 min, followed by a more rapid increase in cell density at 180 min. The most plausible interpretation of this finding is that the first 60 min involved gradual cell attachment from the existing cells in the suspension, while the following 120 min involved the proliferation of the cells in suspension as well as the cells adhering to the surfaces. In support of this, *S. aureus* has been shown to have a doubling time in the 107 – 140 min range in tryptone soy broth (TSB) (Louch et al. 1997). During the first 60 min of incubation, cell density was not significantly different on any of the 4 modified surfaces (P > 0.05). After 180 min of incubation, there were minor differences in cell density on the four surfaces – however, these results did not display a relationship with the surface electrostatic charge. This is interesting because the existing literature is inconsistent with respect to whether and to what degree surface electrostatic charge influences bacterial cell attachment. While some studies report that negatively charged surfaces are inherently repellent to bacteria, the reality of the matter appears much more complicated (Kreve and dos Reis 2022). It involves many variables related to surface properties, the ionic and molecular constituents of the media, and the characteristics of the bacterium. It is well established that *S. aureus* has a net negative cell surface charge due to the presence of negatively charged surface components. It is also well documented that *S. aureus* can actively modify its surface structures to modulate charge by D-alanylation of teichoic acids and lysylation of phosphatidylglycerol (Weidenmaier et al. 2005). Our group recently reported that when *S. aureus* is attached to an unmodified titanium surface, the *dlt* operon and *mprF* gene are upregulated, reducing the net negative charge (Hayles et al. 2024). Similarly, another group reported the upregulation of the same genes upon attachment to a polyethylene terephthalate (PET) surface (Tomlinson et al. 2021). This is intuitive because both titanium and PET possess a net negative charge. Thus, by reducing its cell-surface negativity, *S. aureus* can minimize repulsive forces to maximize attachment to these substrates. In the present study, the rate of attachment of *S. aureus* to negatively charged surfaces was approximately equal to that on more positively charged surfaces. Together, these data support the hypothesis that *S. aureus* can adapt to different material electrostatic properties to maximize its attachment to the material surface.

Our attachment assay on the four plasma-polymer-modified substrates investigated whether the rate of attachment could be correlated with the material ζ-potential. Having observed that there was no discernible correlation between electrostatic charge and rates of cell attachment, we inferred that *S. aureus* was actively responding to the variations in surface electrostatic charge. To further characterize this phenomenon, we narrowed our focus to AA (+ 5 mV) and AC (−79 mV) surfaces for the remainder of the studies since these were the two most positively and negatively charged surfaces, respectively.

### Expression of genes related to cell surface charge

**Fig. 3:**
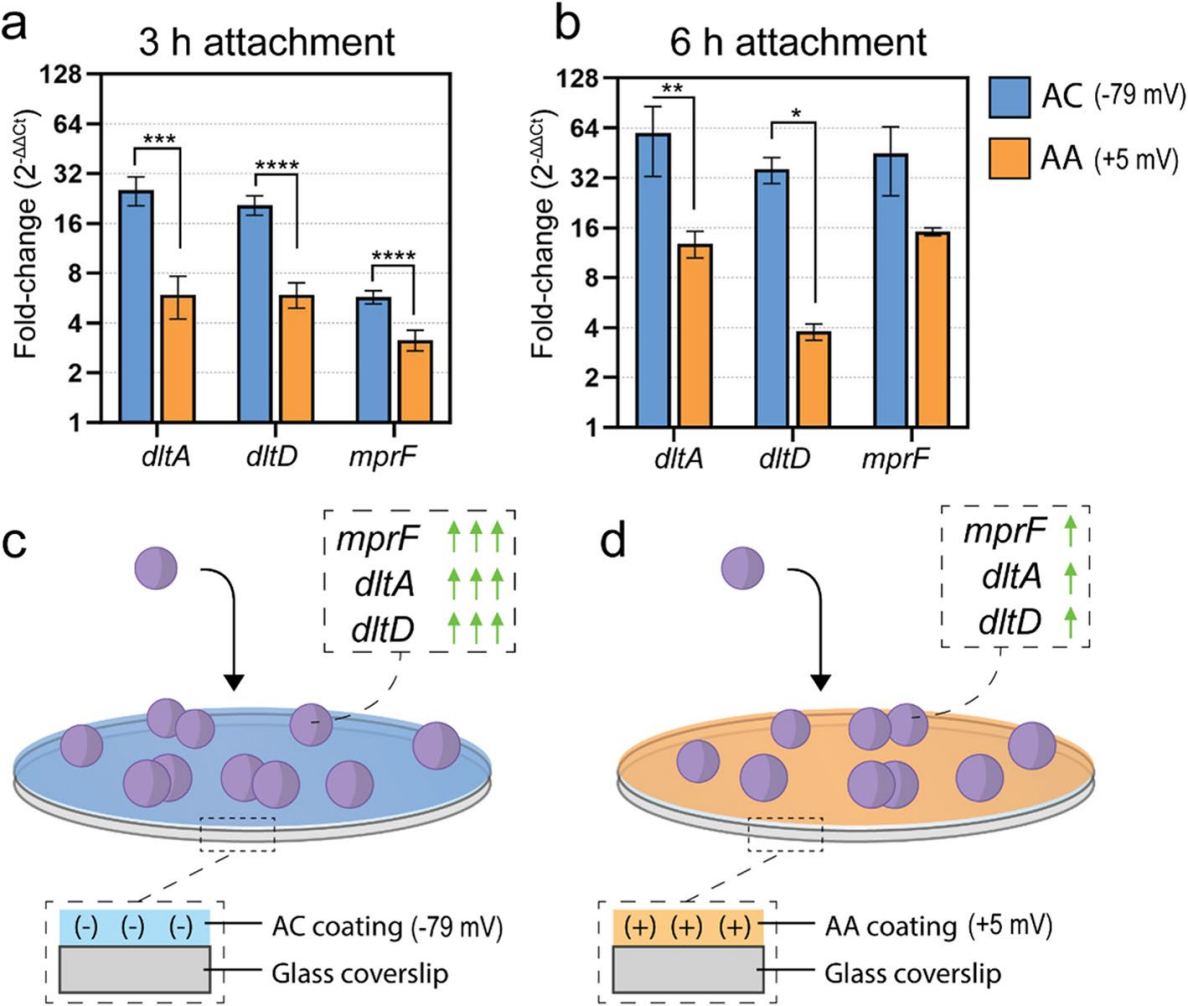
Expression of genes associated with surface charge modifications of *S. aureus.***a** and **b)** Fold change of genes responsible for cell surface charge modifications, measured after 3 and 6 h of attachment to the two charged surfaces. Quantitative RT-PCR measured the expression, and the fold-change was reported as a comparison between surface-attached cells and planktonic cells. **c** and** d**) Schematic representation of the interaction between charged surfaces and *S. aureus*, showing the influence on relative gene expression. Data plotted as mean ± SD, *n* = 3, * *P* < 0.05, ** *P* < 0.01, *** *P* < 0.001 and **** *P* < 0.0001

Our attachment studies suggested that *S. aureus* adapts its surface properties to maximize its attachment to differently charged surfaces. To further investigate this possibility, we measured the expression of genes known to modify cell surface charge – namely, the *dlt* operon (represented here by *dltA* and *dltD*) and *mprF*. We attached *S. aureus* to the charged surfaces for 3 or 6 h, then detached the cells, immediately extracted the RNA, and then used quantitative RT-PCR to determine the degree of expression of these genes (Fig. 3). We observed an increase in expression for all genes on both surfaces, compared to that in free-floating planktonic cells. As expected, the measured genes were more strongly upregulated on the negatively charged AC surface than on the positively charged AA surface (P < 0.0001). A plausible explanation for this can be presented with the following two assertions: 1) these genes play a role in the process of biofilm formation and are thus upregulated as a general response during cell attachment, and 2) due to the influence of these genes on cell surface charge, their degree of expression is governed by the electrostatic properties of the material to which the cell is attaching. In support of the former, another group reported an upregulation of *dlt* and *mprF* after 5 h incubation on polyethylene terephthalate (PET) (Tomlinson et al. 2021). Similarly, we previously reported that these genes were upregulated on *S. aureus* attached to titanium (Hayles et al. 2024). Both PET and titanium have a negative surface charge. It should be noted that herein, we report for the first time direct measurements of the expression of the *dlt* operon or *mprF* in *S. aureus* attached to positively charged surfaces.

During the initial stages of biofilm formation, bacteria secrete extracellular polymeric substances (EPS) to promote the process. One component of EPS is extracellular DNA (eDNA), which possesses a negative charge. It has been reported that the electrostatic properties of eDNA enhance the structural integrity of biofilms by acting as a bridge in cell–cell and cell–matrix interactions (Dengler et al. 2015). Similarly, cationic virulence factors of *S. aureus* have been reported to play a role in the electrostatic environment of biofilm (Graf et al. 2019). These examples support the assertion that *S. aureus* can actively modulate the electrostatic properties of its local environment for biofilm formation. Building from this understanding, it is conceivable that *S. aureus* regulates its expression of the *dlt* operon and *mprF* as a targeted response to the specific electrostatic properties of the material substrate to which it attaches. The different expression levels of these genes on our positively and negatively charged surfaces are indeed strong support for that interpretation.

### Synchrotron-sourced ATR-FTIR analysis of *S. aureus* on charged substrates

**Fig. 4:**
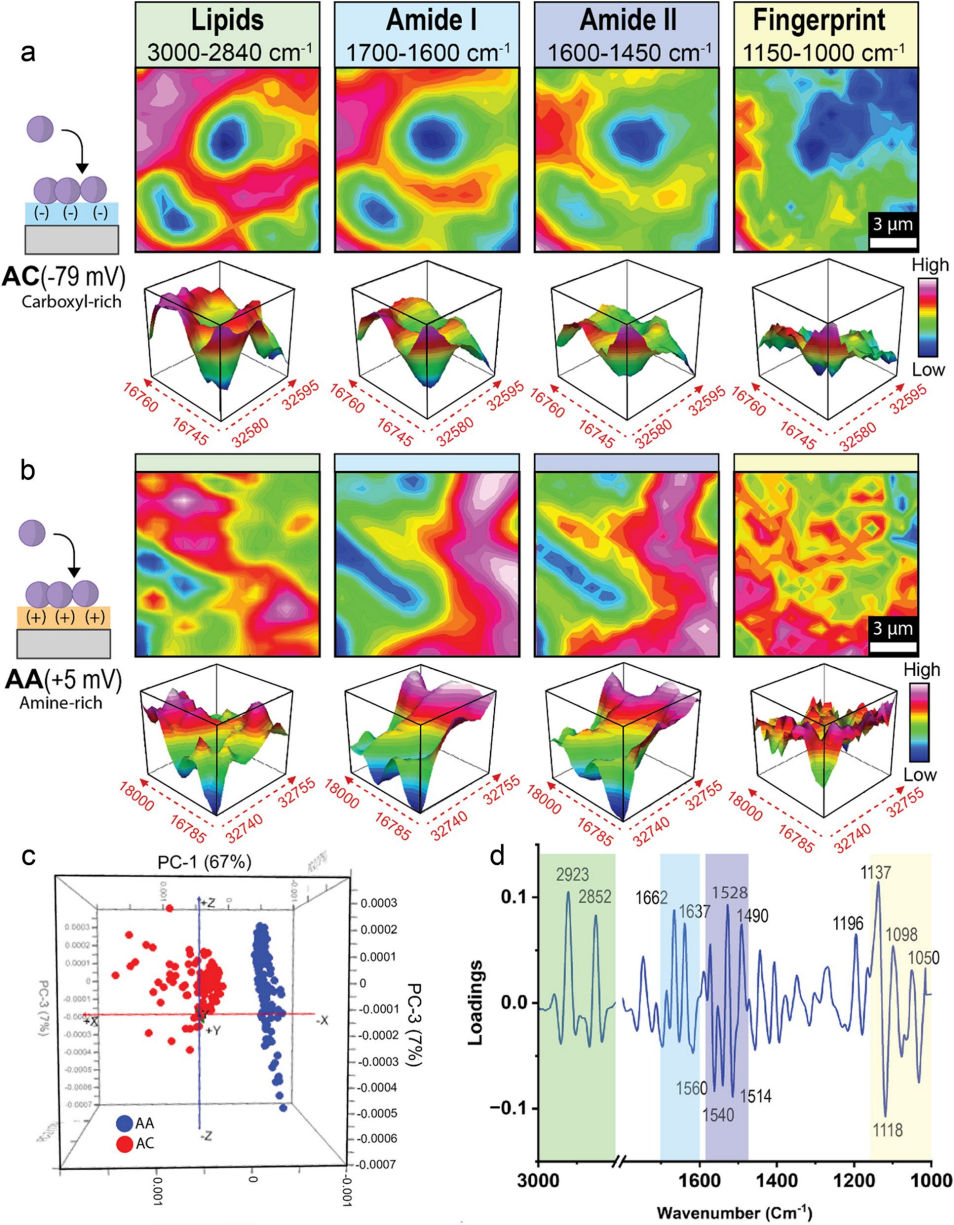
Synchrotron-sourced attenuated total-reflectance Fourier-transform infrared spectroscopy analysis of *S. aureus* attached to AC (carboxyl-rich, −79 mV) and AA (amine-rich, + 5 mV) surfaces. **a** and **b**) Heatmaps of the absorption intensity of the 4 biologically relevant spectral ranges of *S. aureus* attached for 6 h to either AC or AA, respectively. **c)** Comparison of loading plots of *S. aureus* attached to AC and AA. **d** HCA comparison of *S. aureus* attached to AC and AA

Subsequently, synchrotron-sourced attenuated total reflectance (ATR) FTIR microspectroscopy was utilized to further characterize the biological response of *S. aureus* attached to positively and negatively charged materials (Fig. 4). This non-destructive FTIR technique is widely used for obtaining biomolecular information without affecting biological structure or functional properties (Penman et al. 2024; Pham et al. 2023). In this study, our analysis focused on the biologically relevant spectral regions, including lipids (3000–2840 cm^−1^), proteins (1700–1600 cm^−1^ for amide I and 1600–1450 cm^−1^ for amide II), and the fingerprint region (1150–1000 cm^−1^), which predominately captures both polysaccharides and nucleic acids. The average second derivative spectra (Fig. S5) reveal appreciable differences in peak intensities within each of these biological regions, indicating that the difference in material surface charge has a significant influence on the biomolecular properties of *S. aureus*. A schematic workflow of the synchrotron-basd analysis is presented in Fig S6.

Figure 4 (a and b) shows heatmaps of absorption intensities across these four biologically relevant spectral regions, providing a qualitative overview of the biomolecular differences influenced by the surface chemistry of the charge-modified substrates. By comparison, the heatmaps from the fingerprint region displayed the most significant variation in absorbance intensity between cells attached to positively charged AA versus negatively charged AC surfaces. Specifically, the absorbance in this region was visually more intense for cells on the amine-rich AA surface. Given that the absorptions in the fingerprint region occur mainly due to polysaccharides and nucleic acids, this suggests an increase in the secretion of extracellular DNA (eDNA) and biofilm-related polysaccharides (such as glycocalyx) in the cells that were attached to the positively charged amine-rich AA surface. This may be interpreted as a directed response of the pathogen to improve its electrostatic attraction to the positively charged AA surface using eDNA and biofilm-related polysaccharides, which possess a negative charge. This is in agreement with published studies that reported that bacteria actively manipulate their electrostatic environment,(Graf et al. 2019) further supporting our FTIR data.

Additional insights into the biomolecular differences in *S. aureus* attached to oppositely charged surfaces can be gained by examining the heavily loaded peaks presented in the PCA loading plot (Fig. 4d), which are the key biomolecular makeup that influences the separation of the two cell groups seen in the PCA score plot in Fig. 4c. In the lipid-associated spectral region (3000–2800 cm^−1^), two prominent negative loadings were observed at 2923 and 2852 cm^−1^, attributable to asymmetric and symmetric ν(C-H) stretches of methylene (-CH_2_) groups of lipids, respectively. This suggests that material electrostatic properties influence the composition of the bacterial membrane. This is logical because the cell envelope composition is known to be highly adaptable and modular depending on changing environmental conditions (Willdigg and Helmann 2021).

Two significant negative loadings were identified in the amide I region towards the positively charged AA coating at 1662 and 1637 cm^−1^, which are characteristic of proteins α-helix and anti-parallel β-sheet, respectively. On the other hand, cells attached to the negatively charged AC surface showed more substantial protein loadings in the amide II range at 1560 and 1514 cm^−1^, representing specific perpendicular and parallel modes of α-helix protein structures, respectively. Together, the loadings in these amide bands indicate conformational alterations in cell envelope proteins between the two groups (Shivu et al. 2013), which is intuitive due to the understanding that protein folding is governed by electrostatic interactions (Kang and Kini 2009).

The ATR-FTIR data can also be leveraged to validate our gene expression analysis findings. On the negatively charged AC surface, we observed a substantial increase in the expression of both *mprF* and the *dlt* operon. The product of *mprF* acts to synthesize lysyl-phosphatidylglycerol, while the *dlt* operon attaches D-alanine to teichoic acids by an ester bond. These molecular changes are reflected in the ATR-FTIR spectral data (Table 2), where we observed stronger peaks corresponding to these markers on the negatively charged AC surface compared to the positively charged AA surface. This supports the hypothesis that negatively charged surfaces trigger *S. aureus* to regulate the electrostatic properties of its cell envelope. A comprehensive list of major peaks and their assignments can be found in Table 2.

**Table 2:** Key wavenumbers, chemical bond allocation, and references

Representative biomolecular component	Vibrational modes	Expected spectral range	Observed peaks (cm^−1^)
Lipids	ν(C-H) stretch	3000–2840 (Böcker et al. 2007)	2923, 2852
Proteins	ν(C = O) stretch (amide I) ν(N–H) deformation and ν(C-N) (amide II)	1700–1600 (Böcker et al. 2007) 1600–1450 (Böcker et al. 2007)	1662, 1637 1560, 1540, 1528, 1514, 1490
Polysaccharides and nucleic acids	ν(C-O) stretch and δ(C–O) deformation (1° and 2°alcohols)	1150–1000 (Movasaghi et al. 2008)	1137, 1118, 1098, 1050
Lysyl-phosphatidylglycerol	ν(C-N) stretch (1° amine) ν(N–H) bend (amines)	1250–1020 (Merck 2022) 1650–1580 (Merck 2022)	1196, 1137, 1118, 1098, 1050 1637
D-alanylated teichoic acid	νs(C–O–C) stretch (ester)	1210–1163 (Merck 2022)	1196

### Antibiotic tolerance of *S. aureus* attached to positively and negatively charged substrates

**Fig. 5:**
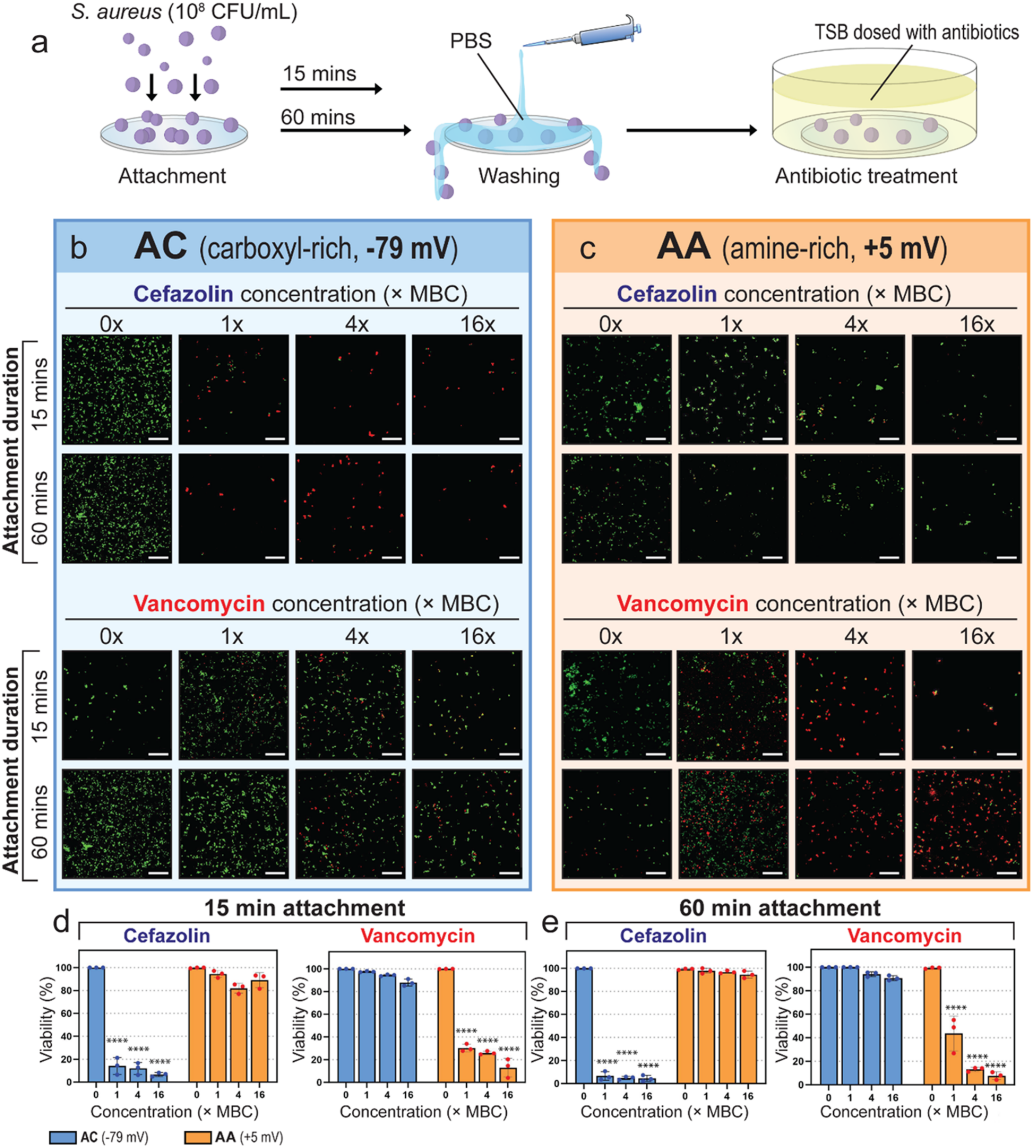
The activity of oppositely charged antibiotics (i.e. cefazolin = −1.0 *e* and vancomycin = + 0.9 *e*) against *S. aureus* attached to charged surfaces. **a** Schematic of the attachment and antibiotic treatment protocol. **b** and **c** Fluorescence micrographs of *S. aureus* following antibiotic treatment on AC and AA-coated samples, respectively. **d** and **e** The quantified cell viability of *S. aureus* following different attachment times and antibiotic treatments. The scale bar represents 40 µm. Data plotted as mean ± SD, *n* = 3 and **** *P* < 0.0001

Next, we investigated whether adhesion to differently charged substrates influences the efficacy of charged antibiotics against *S. aureus *(Fig. 5 and Table S3). To do this, we chose the two surface coatings with the most negative (AC, −79 mV) and most positive (AA, + 5 mV) ζ-potentials from our panel of plasma-polymer coatings. We then selected two antibiotics, cefazolin and vancomycin, since these are commonly used prophylactically in surgical procedures (Shahi and Parvizi 2015). At physiological pH, cefazolin was calculated to have an average molecular charge of −1.0 *e* (elementary charge, equivalent of 1.6 × 10^–19^ coloumbs), while vancomycin has a charge of + 0.9 *e*, due to the titratable groups associated with the two compounds (Fig. S7). These two antibiotics were also selected because their site of activity (peptidoglycan) is within the cell envelope, and thus they would be exposed to the electrostatic microenvironment of the cell envelope.

Indeed, the efficacy of cefazolin and vancomycin against early adherent cells (15- and 60-min attachment) was strongly influenced by the substrate to which the pathogen was attached. In choosing antibiotic concentrations, we first established the minimum inhibitory and minimum bactericidal concentration (MIC and MBC) against planktonic cells (Fig. S8). Then, to test the antibiotic performance on surface-attached cells, we utilized concentrations equivalent to MBC and greater (1 × , 4 × and 16 × MBC) (Fig. 5). On the negatively charged AC surface, cefazolin remained highly effective while vancomycin was virtually ineffective. With 15 min of pre-treatment attachment, cefazolin reduced the cell viability to 14, 12 and 7% at concentrations equivalent to 1 × , 4 × and 16 × MBC, respectively (Fig. 5d). When the pre-treatment duration was increased to 60 min, cefazolin reduced the viability of *S. aureus* to approximately 11, 5 and 2% at concentrations equivalent to 1 × , 4 × and 16 × MBC, respectively. Analysis of the fluorescence micrographs taken under these conditions (Fig. 5b, top) shows a notable decrease in the number of cells per image, indicating that the cefazolin treatment on the AC surface could kill existing cells while inhibiting the division of new cells. When we used vancomycin against *S. aureus* attached to the negatively charged AC surface, we observed that the antibiotic-exposed cells were just as abundant as the non-treated cells, indicating a lack of efficacy (Fig. 5b, bottom). Furthermore, the viability of *S. aureus,* following vancomycin treatment on the AC surface, was approximately 96, 95 and 91% at concentrations equivalent to 1 × , 4 × and 16 × MBC, respectively. Comparably poor activity of vancomycin was also noted with 60 min of pre-treatment attachment (Fig. 5e).

In contrast, when the positively charged AA surface was used as the material for cell attachment, we observed a reversal of these trends. When *S. aureus* was attached for 15 min to the positively charged AA surface and treated with cefazolin, there was virtually no bactericidal activity, as indicated by a predominant green fluorescence in the cells (Fig. 5c, top). On the AA surface, *S. aureus* maintained a high viability between approx. 85 and 94% following cefazolin treatment (when given 15 min of pre-treatment attachment), or approx. 98% (when given 60 min of pre-treatment attachment). When we substituted vancomycin for cefazolin and incubated *S. aureus* for 15 min on AA prior to antibiotic treatment, we observed the cell viability reduce to approximately 30, 26 and 13% at concentrations equivalent to 1 × , 4 × and 16 × MBC, respectively. When given 60 min of pre-treatment attachment, vancomycin reduced the *S. aureus* viability to approximately 44, 13 and 8% at concentrations equivalent to 1 × , 4 × and 16 × MBC, respectively.

The results of these experiments highlight the strong influence of material surface properties on the activity of different clinically relevant pathogens. We designed the antibiotic sensitivity studies to mimic the early attachment period during which the effects of surgical antibiotic prophylaxis are most pertinent. To further characterize this phenomenon, we adapted the study to incorporate more extended periods of cell attachment (3 and 6 h) prior to antibiotic treatment (Fig. S9 and Table S4). The rationale here is that prolonged exposure to the varying electrostatic surfaces may continue to affect the response of *S. aureus* over time, further influencing sensitivity to antibiotics. Following analysis of these increased attachment durations, we observed a trend comparable to that shown for 15- and 60-min attachment durations. In contrast, we observed larger populations of cells in all conditions (Fig S8 b and c), which is expected due to the cells having more time to attach and divide prior to treatment. Interestingly, although we observed a similar trend (where cefazolin was only effective on AC, and vancomycin was only effective on AA), the intensity of this effect was dampened. Specifically, cefazolin could only reduce the viability of *S. aureus* on AC to approximately 20% (with 3 h attachment, 16 × MBC), or approx. 52% (with 6 h attachment, 16 × MBC). Similarly, when *S. aureus* was cultured for extended durations on AA, vancomycin could only reduce the cell viability to approx. 34% (with 3 h attachment, 16 × MBC) or 33% (with 6 h attachment, 16 × MBC). The reduced intensity of the phenomenon observed at 3 and 6 h attachment is likely explained by factors beyond the ones being experimentally manipulated, such as cell number and density and the emerging presence of extracellular polymeric substances, which can act as a penetration barrier to antimicrobials (Singh et al. 2010). This suggests that to take full advantage of the electrostatic environment at the pathogen-material interface, it is most preferable to deploy antibiotics at the earliest time during attachment – such as in the clinical scenario of prophylactic administration of antibiotics immediately before implant placement surgery.

**Fig. 6:**
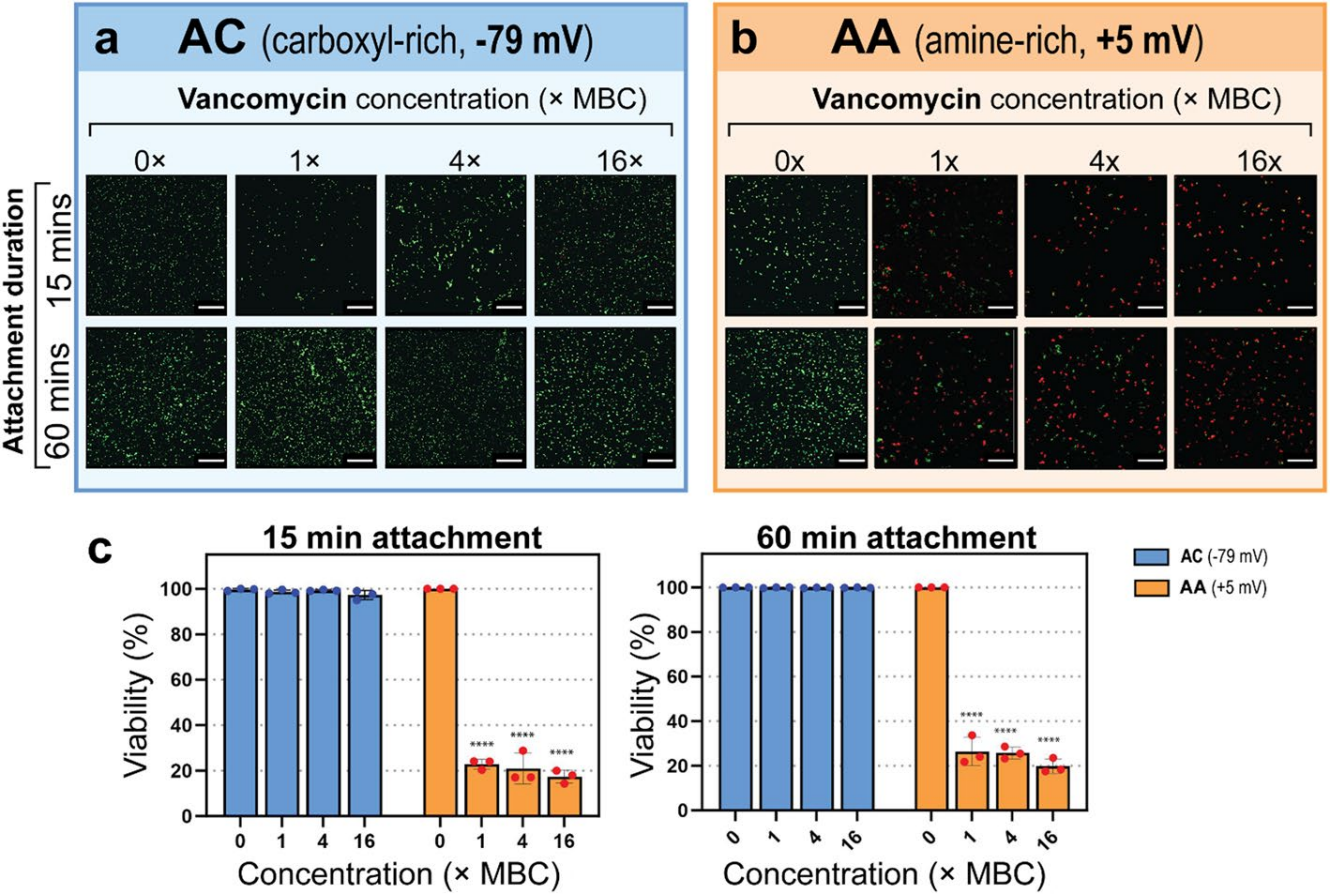
The activity of charged antibiotics against *S. aureus* (MRSA) attached to charged surfaces. **a** and **b** Fluorescence micrographs of *S. aureus* ATCC700699 following antibiotic treatment on AC and AA-coated samples, respectively. Scale bars represent 40 µm. **c** and** d** Bar charts showing the cell viability of *S. aureus* following antibiotic treatment after different pre-treatment attachment durations. Data plotted as mean ± SD, *n* = 3 and **** *P* < 0.0001

To investigate whether this phenomenon was strain-specific or more broadly applicable, we repeated the study using a multidrug-resistant strain of *S. aureus* (MRSA). To maintain consistency with our previous experiments, we investigated the phenomenon during the early stages of cell attachment (15- and 60-min pre-treatment attachment, Fig. 6and Table S5) and later stages (3- and 6-h pre-treatment attachment, Fig S10 and Table S6). The primary defining characteristic of MRSA is its alternatively structured penicillin-binding protein (PBP2a), which is structurally modified from the native protein (PBP) (Lim and Strynadka 2002). The structural difference of PBP2a is within its binding site, enabling MRSA to resist all β-lactam based antibiotics such as penicillin, methicillin or cefazolin. It is therefore not surprising that cefazolin displayed no antibacterial activity on either surface against MRSA (Fig. S11). However, as *S. aureus* (MRSA) remains sensitive to vancomycin, we observed a trend similar to what was shown for the drug sensitive *S. aureus* in Figs. 5 and S7. Specifically, vancomycin was ineffective on the negatively charged AC surface, regardless of attachment duration. In contrast, on the positively charged AA surface, vancomycin reduced the cell viability to approximately 20–25% between 1–16 × MBC for both 15- and 60-min pre-treatment attachment. Again, like the drug-sensitive isolate, *S. aureus* (MRSA) became more tolerant of vancomycin when it had a longer time to attach and mature (3- and 6-h pre-treatment attachment). Specifically, with 3 h attachment, vancomycin reduced the cell viability to approx. 77, 69 and 34%, at concentrations equivalent to 1 × , 4 × and 16 × MBC, respectively. Comparable observations were made when the cells had 6 h of pre-treatment attachment, with reduced viability measured at 69, 54 and 22%, at concentrations equivalent to 1 × , 4 × and 16 × MBC, respectively. Again, this suggests that after 3 h of attachment, factors beyond the initial electrostatic interactions become more important. The significance of this finding is that the phenomenon described in this study is not exclusive to the typical laboratory strain of *S. aureus,* and that even in the case of multi drug-resistant *S. aureus* it is still possible to maximize activity of vancomycin by careful consideration of the electrostatic interface between the pathogen and material.

**Fig. 7:**
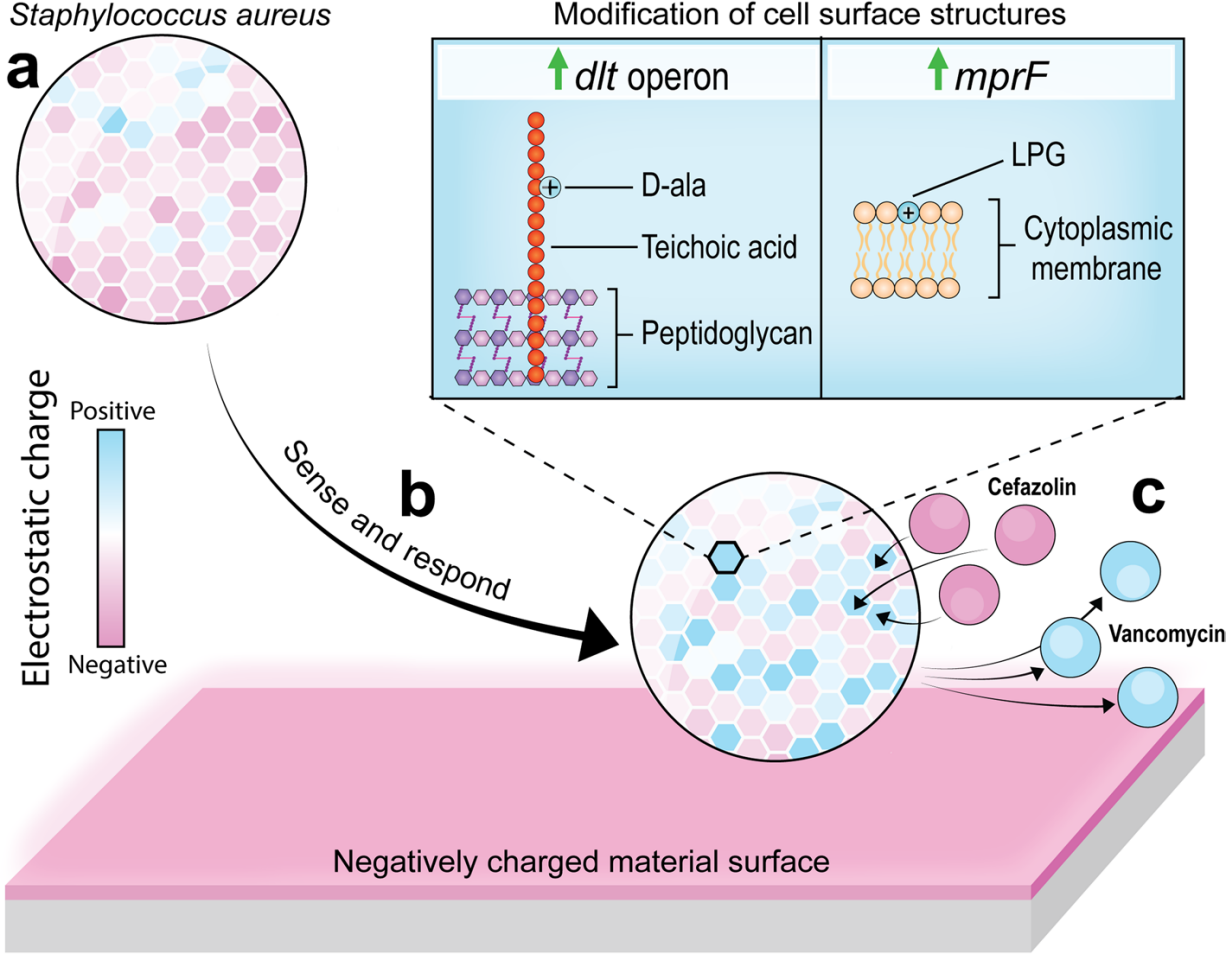
Schematic representation of the proposed phenomenon. **a** a planktonic *S. aureus* cell is depicted with colored regions representing areas with differing cell surface charges. The net surface charge is strongly negative. **b** the cell senses and responds to the negatively charged material surface by upregulating the *dlt* operon and *mprF* to shift its surface charge in the positive direction. **c** the shift in cell surface charge causes a differential response to cefazolin and vancomycin. Blue shading represents a positive charge, while pink shading represents a negative charge

The results presented in this study draw attention to a significantly underappreciated phenomenon that occurs at the biomaterial-pathogen interface. Specifically, we revealed that the envelope of *S. aureus* undergoes phenotypic changes in response to differently charged material surfaces. This interaction selectively influences the activity of differently charged antibiotics. An illustration that visualizes the proposed phenomenon is provided in Fig. 7. To the best of our knowledge, this is the first report to draw a connection between the physicochemical properties of materials and the outcomes of antibiotic treatment against adherent cells. Nevertheless, support for our interpretation of this phenomenon can be found throughout the existing literature. One report showed that adherent staphylococci are more tolerant to the positively charged antibiotic daptomycin in a biofilm-independent manner; i.e., the effect could not be attributed to the presence of EPS (John Anne et al. 2011). As such, the authors speculated that the increased tolerance was driven by a reduction in cell surface negativity. This is clearly in agreement with our results and interpretation; however, the authors did not study the biological response of the pathogen to materials with different charges. Instead, the report attributed the increased daptomycin tolerance to phenotypic changes that universally occur in response to surface attachment.

Similarly, numerous reports connect the function of the *dlt* operon and *mprF* to enhanced tolerance and resistance against positively charged antimicrobials, such as daptomycin (Bayer et al. 2013) and vancomycin (Ruzin et al. 2003). In some cases, resistance to positively charged antimicrobials has been attributed to dysregulation of *mprF* and the *dlt* operon, which both drive a reduction in the negative charge of the cell surface (Bayer et al. 2016). However, these charge-modifying systems have not previously been linked to the ability of bacteria to sense and respond to material surface charge for the purpose of attachment. Nevertheless, it is within reason to assert that charge modification systems facilitate attachment by responding to the physicochemical properties of material surfaces because other similar sense-and-respond systems have been reported (Tuson and Weibel 2013).

The findings of this study promise to be impactful in the clinic because they reveal the existence of a phenomenon that may be exploited immediately to improve surgical antibiotic prophylaxis and mitigate infections. Different biomaterials have different physicochemical properties, so antibiotic outcomes may be improved by carefully considering the material properties on a 'personalized' basis. Clinicians, surgeons and their patients stand to benefit from this new understanding, as the optimal strategy for antibiotic prophylaxis may be selected based on its capacity to exploit the emergent weaknesses of adherent pathogens. It is likely that the described phenomenon applies universally across all implant applications, but it should be emphasized that the biomaterial-pathogen interface varies between different implant applications. For example, orthopedic implants are typically titanium-based and commonly compromised by Gram-positive bacteria such as *S. aureus*.(Flurin et al. 2019) In contrast, catheter applications are commonly based on silicone and are frequently compromised by Gram-negative pathogens such as *Escherichia coli. *(Majumder et al. 2018)*. *These two implant applications are expected to have different biomaterial-pathogen interfaces and would, therefore, require uniquely tailored biomaterial-antibiotic pairing strategies based on their specific electrostatic microenvironments. Therefore, this necessitates further research into the properties of specific biomaterial-pathogen interfaces, and this would lead to significant improvements to patient care.

The newly acquired knowledge reported in this paper that biomaterial surface properties influence antibiotic outcomes may represent an open window of opportunity to explore further advanced infection mitigation strategies. In particular, addressing the increasing threat of antibiotic resistance may be possible by carefully tailoring the biomaterial-pathogen interface. This could be possible in cases where the mechanism of resistance is based upon over-active export pumps. By engineering a surface that promotes the influx of antibiotics, it may be possible to outpace the activity of export pumps, thereby nullifying the mechanism responsible for resistance. These hypotheses are worthy of investigation, as they lead to novel and highly valuable strategies to tackle antimicrobial resistance, one of the fastest-growing threats in medicine.

## Conclusion

In the present study, we investigated whether and to what extent the electrostatic surface properties of materials influence the tolerance of *S. aureus* to commonly used prophylactic antibiotics. To do so, we used plasma-polymer deposition to fabricate a panel of model substrates with ζ-potentials ranging from positive (+ 5 mV) to negative (−79 mV). We determined that there was no relationship between the material surface charge and the bacterial attachment capacity in the early stages of cell attachment. This indicated that *S. aureus* actively modulates its cell surface properties to tailor its interactions to materials with different surface potentials. We investigated this further using quantitative RT-PCR to analyze the expression of genes related to cell surface charge, namely *mprF* and the *dlt* operon. These data supported our hypothesis that *S. aureus* adapts its cell surface properties to facilitate attachment to the surface chemistry of the material to which it is exposed. We further verified this using synchrotron-sourced ATR-FTIR microspectroscopy to reveal the biomolecular differences between cells attached to a positive or negatively charged surface. Importantly, we determined how this biological phenomenon influences antibiotic tolerance. We challenged early adherent cells on a positively and negatively charged substrate using two oppositely charged antibiotics (vancomycin and cefazolin), which operate by almost the same mechanism and are active in the same cellular location. The results showed that vancomycin (positively charged) is more active against *S. aureus* attached to a positively charged substrate. At the same time, cefazolin (negatively) is more active against *S. aureus* attached to a negatively charged substrate. This finding is consistent with the gene expression analysis, which showed that the degree of expression of positive-shifting genes (*mprF* and *dlt* operon) was influenced by the positive or negative surface charge of the material. Overall, the results of this study highlight a potentially important phenomenon that clinicians may exploit to maximize the success rate of antibiotic prophylaxis during implant placement surgery.

## Materials and methods

### Plasma polymer deposition

Surfaces with four different chemistries were prepared using a custom-built plasma reactor with a radio frequency (13.56 MHz) plasma generator. 2-methyl-2-oxazoline, allylamine, acrylic acid and 1,7-octadiene were the four monomer precursors (Sigma Aldrich, Missouri, USA) used for the experiment. Before treatment, all the glass coverslips were cleaned by ultrasonication in acetone and Milli Q water and dried with a stream of nitrogen gas. The cleaned glass coverslips were placed on the round bottom electrode of the reactor and the glass chamber was evacuated to a base pressure of 2.3 × 10^−3^ mbar. Initially, the glass coverslips were cleaned using air plasma for 5 min set at 50 W and 1 × 10^−1^ mbar pressure. Then, they were coated separately with each monomer, whose deposition conditions are listed in Table 3. Deposition was carried out at room temperature. The plasma-coated coverslips were immediately placed in 24 well plates and vacuum sealed at room temperature to ensure post-deposition stabilization and kept sealed until further use.

**Table 3:** Plasma deposition conditions using four different precursor monomers

Monomers	Time (min)	Power (W)	Pressure (mbar)	Flow rate (sccm)
1,7-octadiene	2	20	1.3 × 10^–1^	4.94
2-methyl-2-oxazoline	2	50	8 × 10^–2^	3.2
Acrylic acid	2	10	1.3 × 10^–1^	6.1
Allylamine	2	40	1.3 × 10^–1^	6.85

### Surface characterization

#### Coating thickness

The coating thickness was measured by a spectroscopic ellipsometer (SENresearch 4.0, SENTECH Instruments, GmbH, Berlin, Germany). The images and data were processed using SpectraRay/4 comprehensive ellipsometry software (SENresearch 4.0).

#### X-ray photoelectron spectroscopy (XPS)

Elemental and chemical analysis were performed with XPS, and the spectra were recorded on a Kratos Axis Ultra XPS spectrometer (Kratos Analytical Ltd, UK). The spectrometer was equipped with a monochromatic Al source and operated at 15 keV and 15 mA to acquire survey spectra from 0 to 1100 eV for the plasma-deposited glass substrates. The data were analyzed using Casa XPS software (http://www.casaxps.com/).

#### Fourier-transform infrared (FTIR) spectroscopy

FTIR measurement was performed as a secondary measurement of the chemical properties of the plasma-deposited polymers. First, 10 mg of finely ground potassium bromide (KBr) powder was dried in an oven overnight before plasma deposition. The plasma was then deposited in a monolayer form in a customized powder-coating plasma system. The precursor monomers were each individually deposited as described above, and a shaker was used to gently agitate the KBr powder and ensure an even coating on the granules. The FTIR spectra were obtained with a PerkinElmer Spectrum 3 FTIR spectrometer (PerkinElmer, MA, USA).

#### Wettability (WCA) measurement

The wettability of the plasma-modified surfaces was measured using a sessile drop contact angle goniometer (RD-SDM02, RD Support, Scotland, UK). 5 µL of milli-Q water was dropped onto the plasma-modified substrates. An image was captured when the drop contacted the substrate. ImageJ v1.53 analysis software (NIH, USA) was used to measure the tangent of the drop at its three-phase contact point (intersection between solid, liquid and gas).

#### Zeta (ζ) potential

A ZPA 2.0 (Dataphysics, Germany) was used to measure the ζ-potential of the plasma deposited substrates over a pH range of 5.5 – 9. An oscillating flow of KCl_(aq)_ (10^–3^ M) was applied in a narrow gap of approximately 130 µm between two plasma-modified substrates with a defined 10 × 20 mm area. An automated dosing unit adjusted the pH between each reading using stock solutions of 1 M HCl_(aq)_ and 1 M KOH_(aq)_.

#### Atomic force microscopy (AFM)

AFM images were acquired using a Bruker Multimode AFM with a Nanoscope V controller using tapping mode in air, with all parameters, including set-point, scan rate and feedback gains, adjusted to optimize image quality. The amplitude set-points were kept at 80 to 90% of the cantilever-free amplitude to minimize the tapping force. The AFM probes used were Mikromasch HQ: NSC15 Si probes with a nominal spring constant of 40 N m^−1^ and a nominal tip diameter of 16 nm. The scanner was calibrated in the x, y and z directions using silicon calibration grids (Bruker model number VGRP: 10 µm pitch, 180 nm depth, PG: 1 μm pitch, 110 nm depth, Mikromasch model TGZ01: 3 µm pitch, 18 nm depth). All analyses of the AFM images were performed using Nanoscope analysis software version 2. Five 10 × 10 μm images were acquired per sample at distinctly separate locations (i.e., the tip was disengaged from the surface and moved some hundreds of microns in the X and Y directions before re-engaging). Roughness analysis was performed on each AFM image, with the values reported representing the Ra and the Rq (RMS) roughness, with the error in each measurement being the standard deviation. R_a_ and R_q_ are standard analysis methods for reporting surface roughness using AFM, but they differ in their mathematical description of roughness. Where Rq is the root mean square average of the height deviations taken from the mean image data line, and R_a_ is the arithmetic average of the absolute values of the surface height deviations measured from the mean plane. In many instances, they give different answers but often follow similar trends between images, as we observed in our case.

#### Cultures and conditions

*Staphylococcus aureus* ATCC25923 and *S. aureus* (MRSA) ATCC700699 were retrieved from glycerol stock stored at −80 °C. For ATCC25923, a single loop of bacteria was streaked onto tryptone soy agar (TSA; Oxoid, ThermoFisher, MA, USA). For ATCC700699, the TSA agar plate was substituted with vancomycin (4 µg/mL). Plates were incubated overnight at 37 °C. A single colony was transferred from the agar plate to 5 mL of tryptone soy broth (TSB, Oxoid, ThermoFisher, MA, USA), or for ATCC700699 TSB supplemented with vancomycin (4 µg/mL), then incubated overnight at 37 °C. The cell concentration of the overnight culture was measured by optical density at 600 nm (OD_600_) in a spectrophotometer (Nanodrop 2000, Thermo Scientific, MA, USA). Then, the culture was diluted in TSB to an OD_600_ value of 1, previously determined to correspond to a cell density of 1 × 10^9^ CFU/mL.

#### Adhesion assessment

Circular glass coverslips, with or without plasma polymer coatings, were individually placed in the wells of a 24-well plate and sterilized by UV for 20 min. The overnight *S. aureus* culture was further diluted to a cell density of 1 × 10^8^ CFU/mL, and 1 mL of the cell suspension was aliquoted into each of the wells containing glass coverslips. The well plate was placed in a sealed container and kept on an orbital shaker (60 RPM) in a warm room (37 °C) for up to 180 min, with samples being retrieved at 15, 30, 60 and 180 min. The retrieved glass coverslips were gently dipped in phosphate-buffered saline (PBS) to remove non-adherent cells, and the coverslips were then submerged in a solution of safranin (1% v/v) in PBS for 5 min. The coverslips were gently dipped in PBS again to remove excess safranin. The density of the attached cells was observed under a light microscope (Leica DM1000, Leica Camera AG, Wetzlar, Germany) at 100 × magnification. Images were captured for each coverslip at random locations in triplicate. Micrographs were converted to black and white to improve the visibility of the cells. Cells were counted in ImageJ v1.53 (NIH, USA), and the cell density was reported in terms of cells/mm^2^. The cell attachment rate was calculated by dividing the cell density accumulated between each time point by the number of minutes elapsed between each time point; this value was reported as cells/mm^2^/min.

#### RNA extraction

Cells attached to substrate samples were gently rinsed in PBS to remove non-adherent cells and detached using ultrasonication (2 min) followed by vortexing (30 s). The cells were pelleted by centrifuge and resuspended in RNA extraction buffer, supplied with the RiboPure Bacteria RNA kit (Invitrogen, MA, USA). The RNA extraction was carried out according to the manufacturer's instructions. RNA quantity was measured, and RNA quality was validated with a Nanodrop 2000c spectrophotometer (ThermoFisher, MA, USA).

#### RT-PCR

PCR master mixes were prepared using SuperScript III Platinum One-Step qRT-PCR Kit (Invitrogen, MA, USA). Primers were added at a concentration of 10 µM. The primer sequences are listed in Table S2. Template RNA (1 ng) was added to each reaction tube in 1 µL aliquots. No-template controls received 1 µL RNAse-free H_2_O instead of template. Reverse transcription and amplification were carried out in 1 step in a Rotor-Gene Q Thermocycler (version 2.1.0, QIAGEN, Hilden, Germany) with the following program: 3 min at 50 °C, 5 min at 95 °C, 40 cycles of [95 °C for 15 s and 60 °C for 30 s]. The signal was acquired at 60 °C. A melt curve was generated at 1 °C increments between 72 and 95 °C. The amplification specificity was verified by melt curve analysis (Table S2). The expression of genes was normalized to the 16S sequence, and the fold-change was calculated using the Livak method (2^−ΔΔCt^) (Livak and Schmittgen 2001).

#### Synchrotron-sourced attenuated total reflectance Fourier transform infrared (ATR-FTIR) microspectroscopy

Synchrotron-sourced ATR-FTIR microspectroscopy was employed to study the biomolecular differences between *S. aureus* cells attached to the positive (AA) and negative (AC) surfaces (workflow schematic presented in Fig. S5). Prior to the data collection, an overnight culture of *S. aureus* ATCC25923 was diluted to a density of 10^7^ CFU/mL and used to immerse the plasma-modified substrates for 6 h. The samples were then gently rinsed 3 times with sterile PBS to remove non-adherent cells and the remaining culture medium. The samples were then placed on the ATR-FTIR stage on the Infrared Microspectroscopy (IRM) beamline at the Australian Synchrotron (Clayton, Victoria). The mapping measurement was performed using a Bruker Vertex 80v spectrometer coupled with a Hyperion 3000 FTIR microscope and a liquid nitrogen-cooled narrow-band mercury cadmium telluride (MCT) detector (Bruker Optik GmbH, Ettlingen, Germany (Vongsvivut et al. 2019). High-resolution spectral maps were collected on the monolayers of cultured cells within the spectral range of 3400–1000 cm^−1^, using a projected aperture size of 2.5 μm and a 1 μm mapping step size (effective pixel size of 1 μm). After that, the raw spectral maps were processed using chemometric methods, including hierarchical cluster analysis (HCA), and principal component analysis (PCA), to identify differences in biomolecular compositions between cells attached to the two charged surfaces. While HCA was used to discriminate and identify spectral groups representing cell clusters, PCA was used to investigate similarities and differences in biomolecular compositions between the two cell groups that were attached to different charged surfaces.

#### Minimum inhibitory concentration (MIC) and minimum bactericidal concentration (MBC)

The minimum inhibitory concentrations of cefazolin and vancomycin were determined using the broth microdilution method, following the standard parameters set by the Clinical and Laboratory Standards Institute (CLSI) (CLSI 2020). The only adjustment was the culture media, which was TSB instead of Mueller–Hinton broth. Briefly, each antibiotic was serially diluted two-fold into TSB in a 96-well plate to a final well volume of 95 µL. To this, 5 µL of diluted bacterial suspension was added to reach a final cell density of 5 × 10^5^ CFU/mL. The well plate was incubated at 37 °C for 18 h, and then the optical density was read at 600 nm in a Synergy HTX Multimode reader (BioTek, Vermont, USA). To determine the MBC, 10 µL aliquots were taken from the wells corresponding to MIC, 2 × MIC, 4 × MIC, and 8 × MIC and dropped onto a TSA plate. After overnight incubation at 37 °C, the lowest concentration at which no colonies formed was determined to be the MBC.

#### Antibiotic sensitivity of surface-adherent cells

As previously described, *S. aureus* was attached to plasma-modified glass coverslips for 3 or 6 h using a cell density of 1 × 10^8^ CFU/mL. Following cell attachment, the coverslips were gently dipped in sterile PBS to remove loosely bound cells. Coverslips were placed in the wells of a 24-well plate and immersed in TSB supplemented with either vancomycin or cefazolin at concentrations of 1, 4 and 16 × MBC, then incubated at 37 °C on an orbital shaker set to 60 RPM for 24 h. The samples were gently rinsed in PBS and then stained with BacLight Live/Dead (Invitrogen, MA, USA) reagents, with an equal proportion of Syto9 and propidium iodide (1.5 µL/mL in PBS) for 15 min in the dark at room temperature. Samples were immediately imaged with an LSM880 confocal laser scanning microscope (Zeiss, Germany), using excitation and emission wavelengths of 480/500 nm for Syto9 and 490/635 nm for propidium iodide. The quantities of live (green stained) and dead (red stained) cells were quantified using ImageJ v1.53 analysis software by first splitting the color channels into red and green and within each channel using the 'find maxima' tool. The quantities of red and green cells were converted to a percentage of viability by the following calculation: $Viability \left(\%\right)= \frac{green spots}{total spots}\times 100$.

#### Cytocompatibility of THP-1 monocyte-like cells

THP-1 cells were seeded into 96 well plasma-coated tissue culture plates at a concentration of 5 × 10^3^ cells well^−1^ for 72 h at 37 °C and 5% CO_2_ and the growth media was replenished once during this period_._ The growth media was RPMI 1640 supplemented with 10% fetal bovine serum, and 1% (v/v) penicillin–streptomycin. Following incubation, 100 µL of 0.5 mg mL^−1^ MTT reagent (Sigma-Aldrich, MI, USA) was added to each well and incubated for 4 h at 37 °C, 5% CO_2_ and 95% humidity. After the incubation, 100 µL of dimethyl sulfoxide (Sigma-Aldrich, MI, USA) was added, mixed gently, and incubated for 15 min at 37 °C, 5% CO_2_ and 95% humidity. The supernatant was transferred to a clear 96-well plate, and absorbance was read at 590 nm using a FLUOstar Omega (BMG Labtech, Ortenberg, Germany) microplate plate reader.

#### Statistical analysis

Graphical data are presented as the mean and standard deviation and were plotted with GraphPad Prism v9.0.0 (GraphPad Software, California, USA). All experiments were performed with triplicate samples at least two times. The attachment study, Live/Dead and RT-PCR fold change analyses were analyzed using a two-way ANOVA with Šídák's multiple comparisons test. The statistical significance of the differential gene expression analysis was determined with an unpaired T-test. A p-value less than 0.05 was regarded as statistically significant.

## Supplementary Information

Additional file 1. Atomic percentages of plasma-modified surface coatings; FTIR analysis of chemical functionalities on plasma-modified surface coatings; AFM micrographs; THP-1 cytocompatibility by MTT; Comparison of average 2nd derivative spectra of S. aureus attached to AC and AA; workflow for synchrotron ATR-FTIR; molecular structure and charge of cefazolin and vancomycin; primers used in RT-PCR and amplification product melting curves; MIC of cefazolin and vancomycin; antibiotic activity against cells attached for 3 and 6 h; antibiotic activity against MRSA on AA and AC coatings attached for 3 and 6 h; activity of cefazolin against MRSA attached for AA and AC.

## Data Availability

Data will be made available by the corresponding authors upon reasonable request.
