# Supplementary material for: Electrostatic charge at the biomaterial-pathogen interface influences antibiotic efficacy
Source: Adv Biotechnol (Singap). 2025 Apr 3;3(2):10. doi: 10.1007/s44307-025-00061-z (PMC11965051; doi:10.1007/s44307-025-00061-z)
Supplement: Supplementary file 1 — Additional file 1. Atomic percentages of plasma-modified surface coatings; FTIR analysis of chemical functionalities on plasma-modified surface coatings; AFM micrographs; THP-1 cytocompatibility by MTT; Comparison of average 2nd derivative spectra of S. aureus attached to AC and AA; workflow for synchrotron ATR-FTIR; molecular structure and charge of cefazolin and vancomycin; primers used in RT-PCR and amplification product melting curves; MIC of cefazolin and vancomycin; antibiotic activity against cells attached for 3 and 6 h; antibiotic activity against MRSA on AA and AC coatings attached for 3 and 6 h; activity of cefazolin against MRSA attached for AA and AC. [file 44307_2025_61_MOESM1_ESM.docx]

Electrostatic Charge at the Biomaterial-Pathogen Interface Influences Antibiotic Efficacy

Andrew Hayles^1*^, Huu Ngoc Nguyen^1,2^, Markos Alemie^1^, Jitraporn Vongsvivut^3^, Neethu Ninan^1^, Richard Bright^1^, Panthihage Ruvini Dabare^4^, Christopher Gibson^5,6^, Vi Khanh Truong^1,7^, Krasimir Vasilev^1*^

^1^Biomedical Nanoengineering Laboratory, Flinders University, Bedford Park 5042, South Australia, Australia

^2^School of Biomedical Engineering, Faculty of Engineering, University of Sydney, 2050, New South Wales, Australia

^3^Infrared Microspectroscopy (IRM) Beamline, ANSTO ‒ Australian Synchrotron, 800 Blackburn Road, Clayton, Victoria 3168, Australia

^4^Academic Unit of STEM, University of South Australia, Mawson Lakes, South Australia, 5095, Australia

^5^Flinders Microscopy and Microanalysis, Flinders University, Bedford Park 5042, South Australia, Australia

^6^Adelaide Microscopy, The University of Adelaide, Adelaide, South Australia 5000, Australia

^7^Deaprtment of Biomedical Engineering, Healthcare Engineering Innovation Centre, Khalifa University, Abu

Dhabi, United Arab Emirates

(*Corresponding authors: [andrew.hayles@flinders.edu.au](mailto:andrew.hayles@flinders.edu.au), [krasimir.vasilev@flinders.edu.au](mailto:krasimir.vasilev@flinders.edu.au))

Keywords: antibiotic prophylaxis, surface charge, drug tolerance, biomaterials, coating, nanotechnology

**Results and Discussion**

*Atomic percentages*


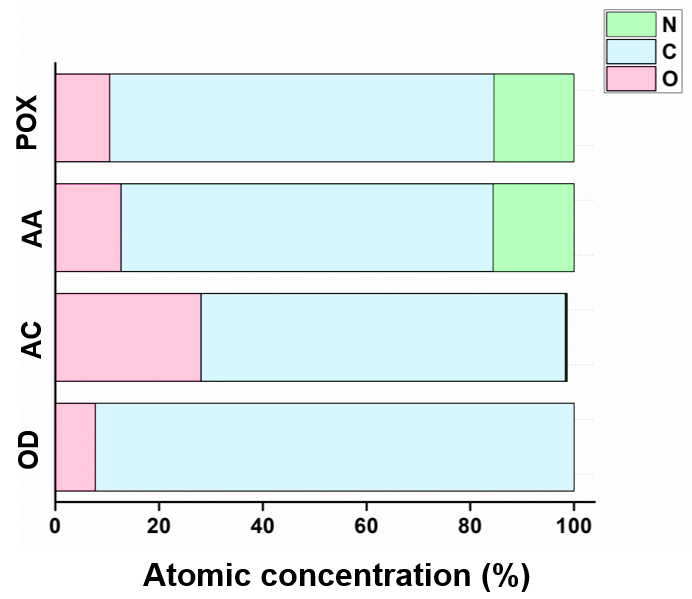


**Fig. S1**. Proportions of N, C and O in the four plasma-modified surfaces.

**Table S1**. Atomic concentrations of N, C and O in the four plasma-modified surfaces

| Plasma-polymer | N (%) | C (%) | O (%) |
| --- | --- | --- | --- |
| POX | 15.4 | 74.1 | 10.5 |
| AA | 15.6 | 71.7 | 12.7 |
| AC | 0 | 71.4 | 28.6 |
| OD | 0 | 92.2 | 7.8 |


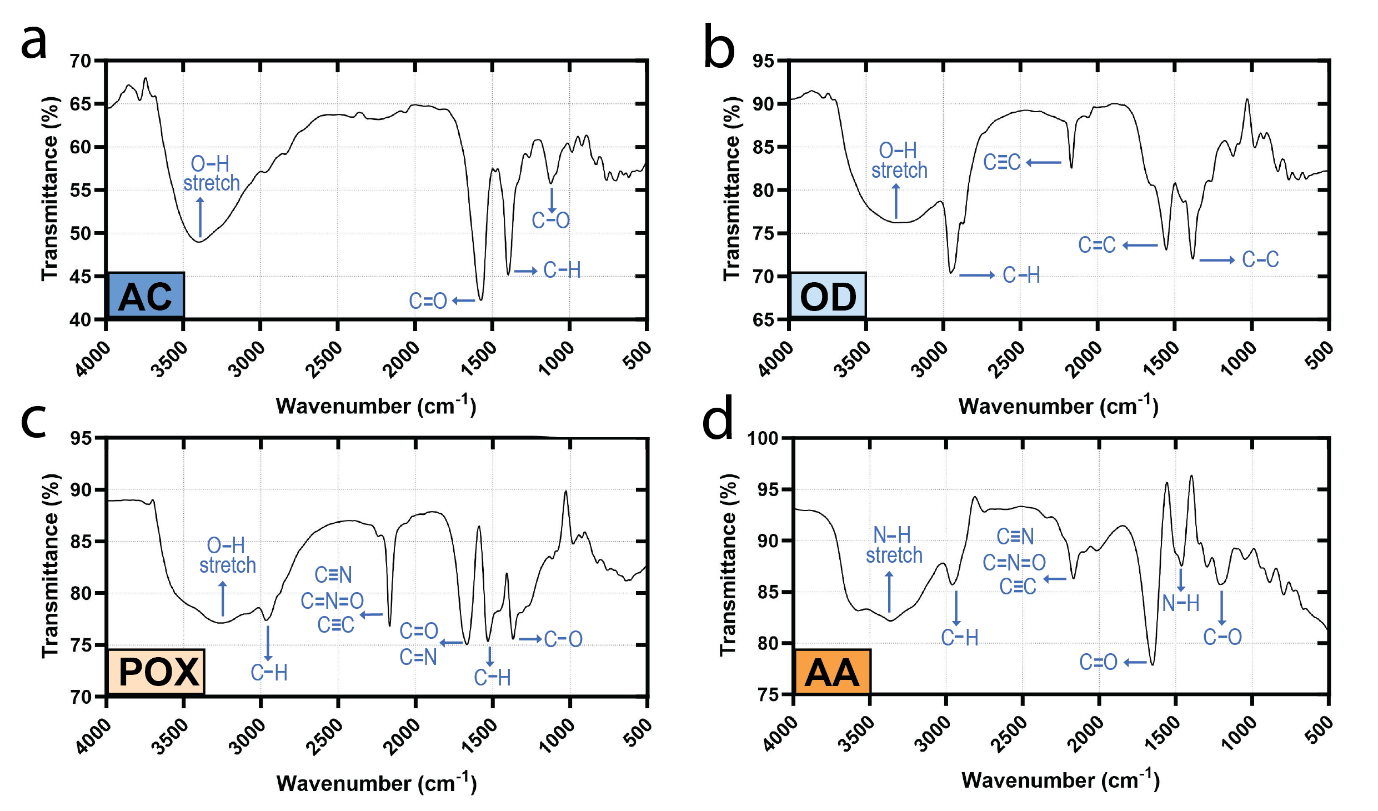


**Fig. S2**. FTIR spectra of the four plasma polymers coated on KBr powder.

*AFM micrographs of four plasma-modified surfaces*


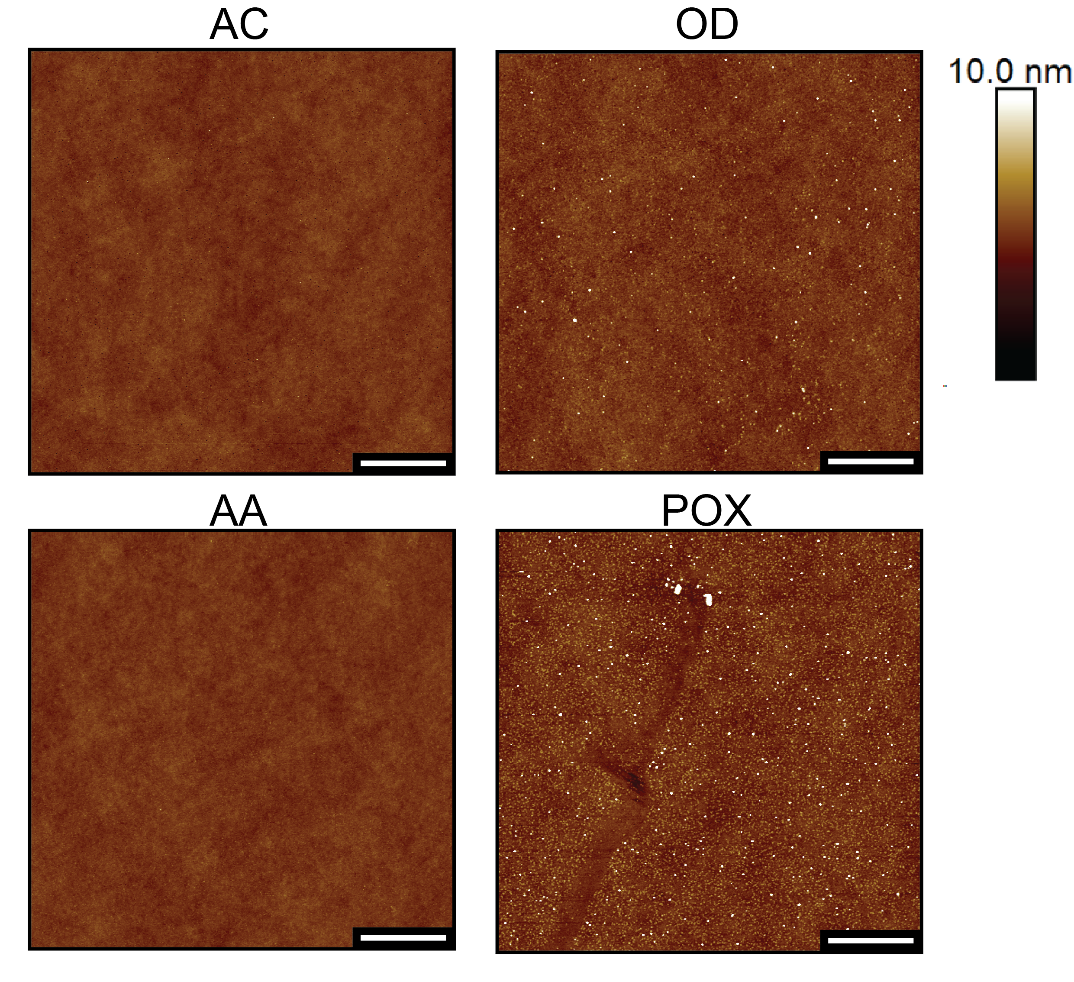


**Fig. S3.** AFM micrographs of the four plasma-modified surfaces. Scale bars represent 2 µm.


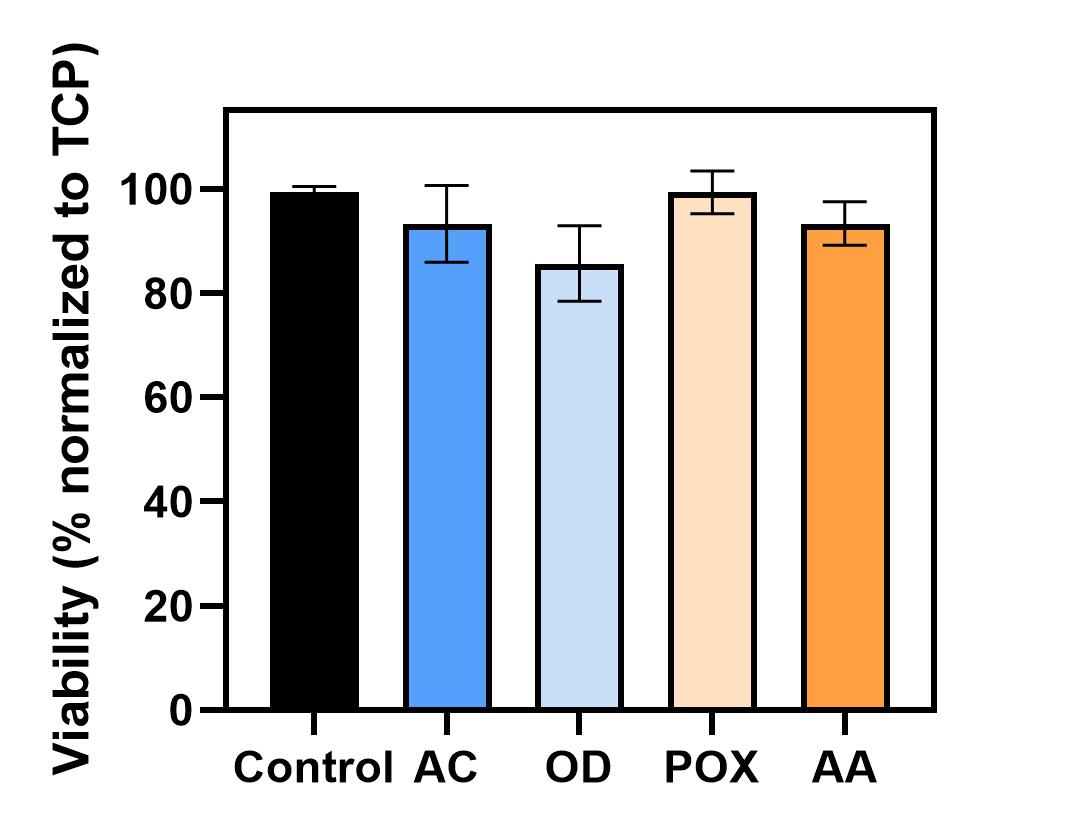


**Fig. S4.** Cytocompatibility of THP-1 cells after 72 h incubation on the four plasma-modified surfaces, as measured by MTT assay.


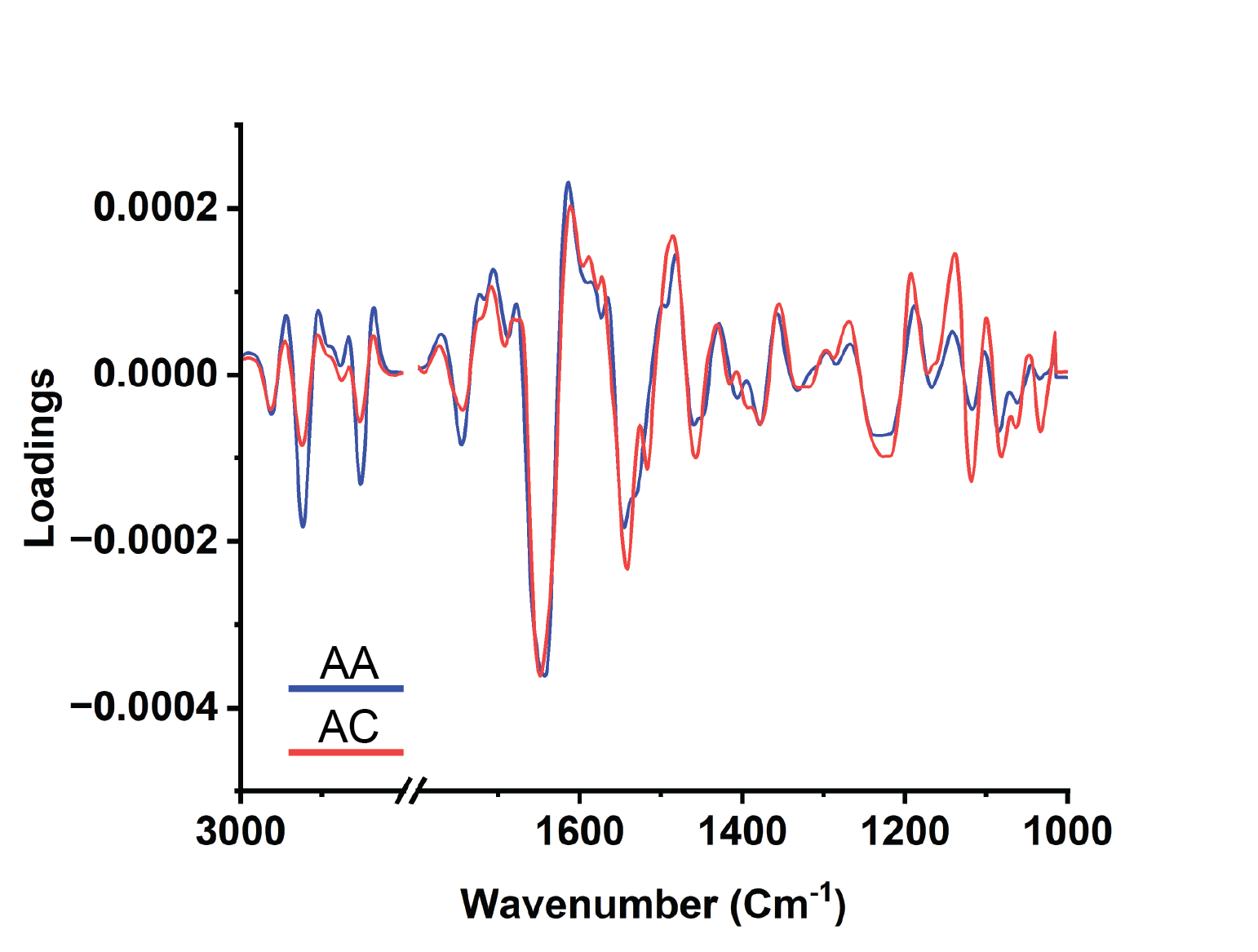


**Fig. S5**. Comparison of average 2^nd^ derivative spectra of *S. aureus* attached to AC and AA

*
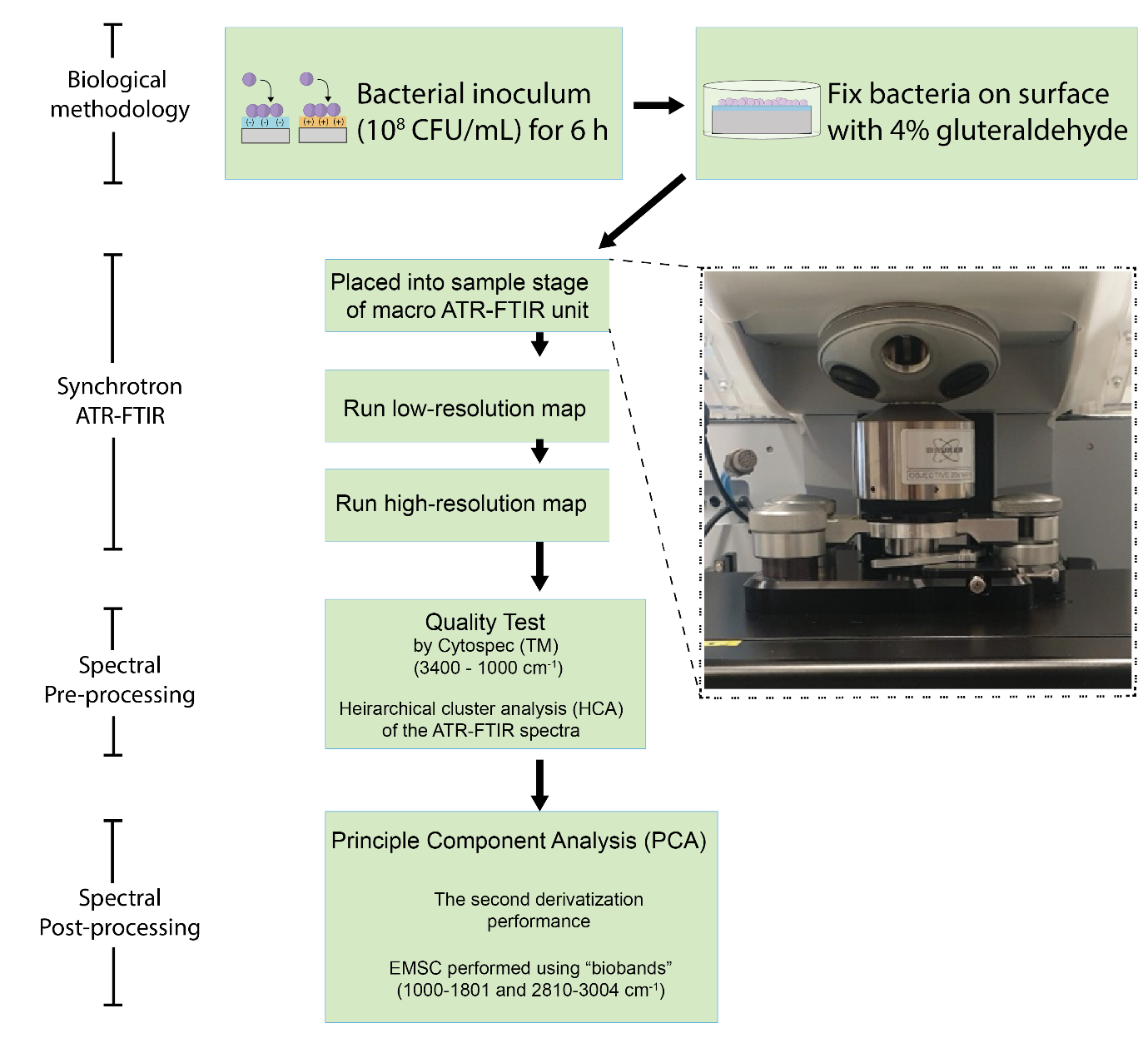
*

**Fig. S6**. Workflow for synchrotron ATR-FTIR analysis

**
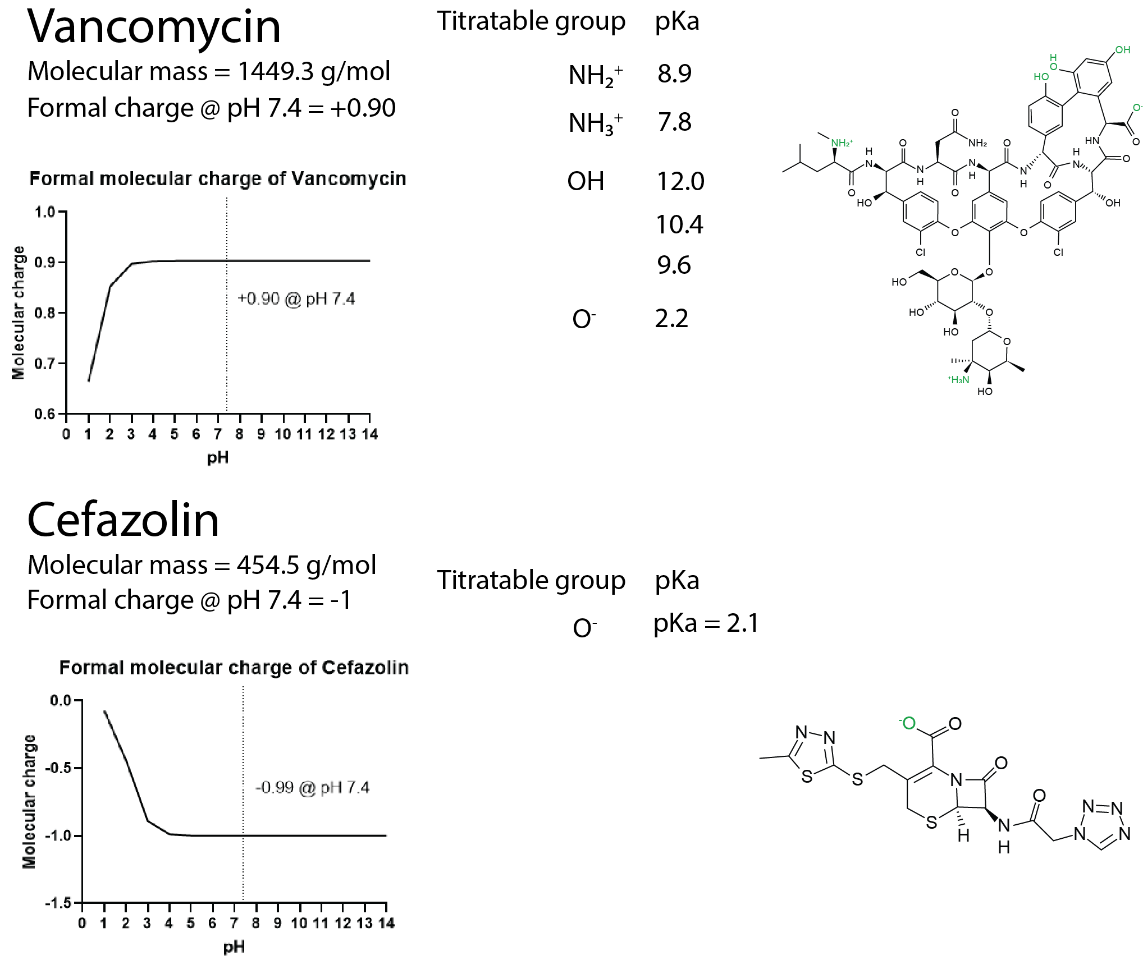
**

**Fig. S7**. Molecular structures of cefazolin and vancomycin. pKa values for titratable groups were sourced from relevant literature. [1, 2]

*Primer validation*

**Table S2.** The primers used in qPCR analysis along with their respective melting curves.

| Gene symbol, primer sequence and reference | Melt curve |
| --- | --- |
| *16S[3]*  F: ACGGTCTTGCTGTCACTTATA  R: TACACATATGTTCTTCCCTAATAA | 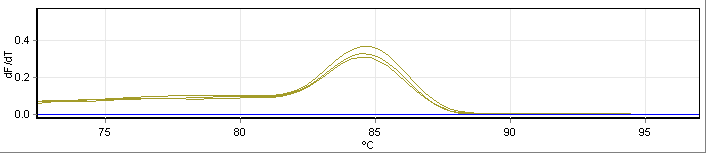 |
| *dltA[4]*  F: CACAGAGCAGCAAAAGCGTTAG  R: ACATATGGTCCAACTGAAGCTACG | 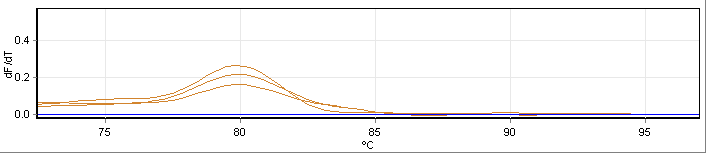 |
| *dltD[4]*  F: TGACCCATTTAATCCTGCAATTG  R: TCTGTAGAACCACCAGCACCTAATAA | 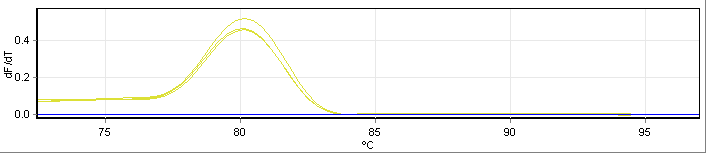 |
| *mprF[5]*  F: TTGTAGGTTTCGGTGGCTTT  R: GATGCATCGAAAACATGGAA | 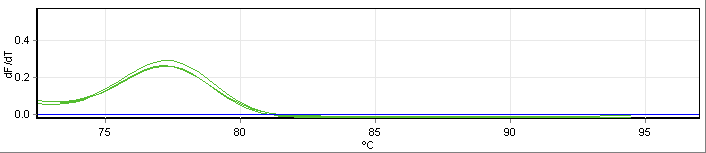 |

*Minimum inhibitory and bactericidal concentrations of antibiotics*

*
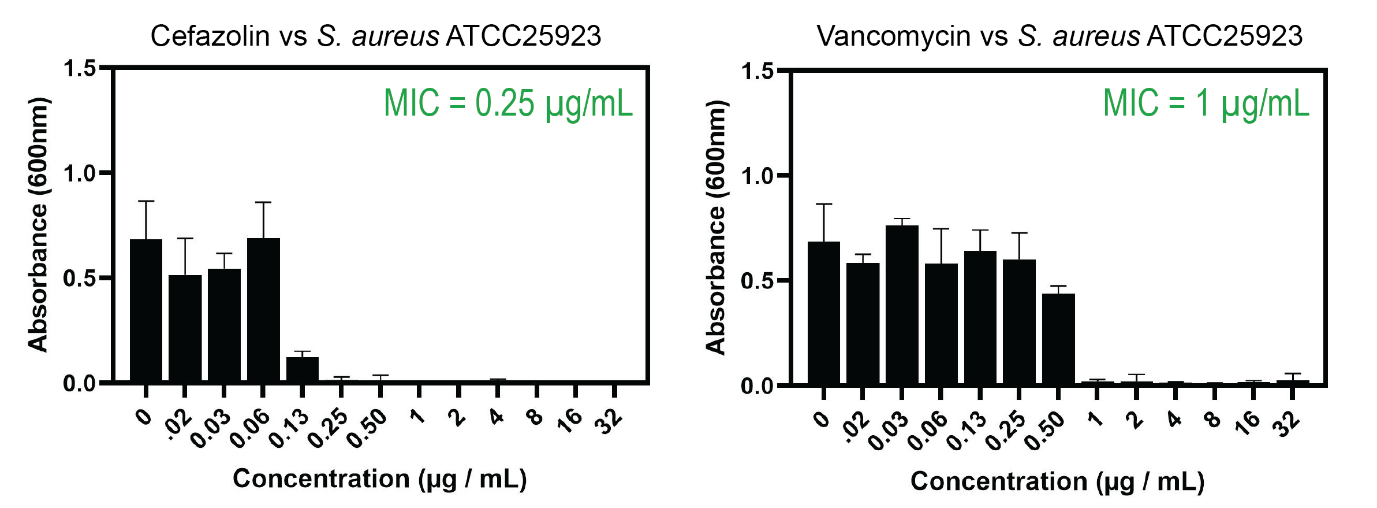
*

**Fig. S8**. The minimum inhibitory concentration of cefazolin and vancomycin against *S. aureus* ATCC25923, as determined by the microdilution method following the standards set by the Clinical and Laboratory Standards Institute (CLSI).[6]


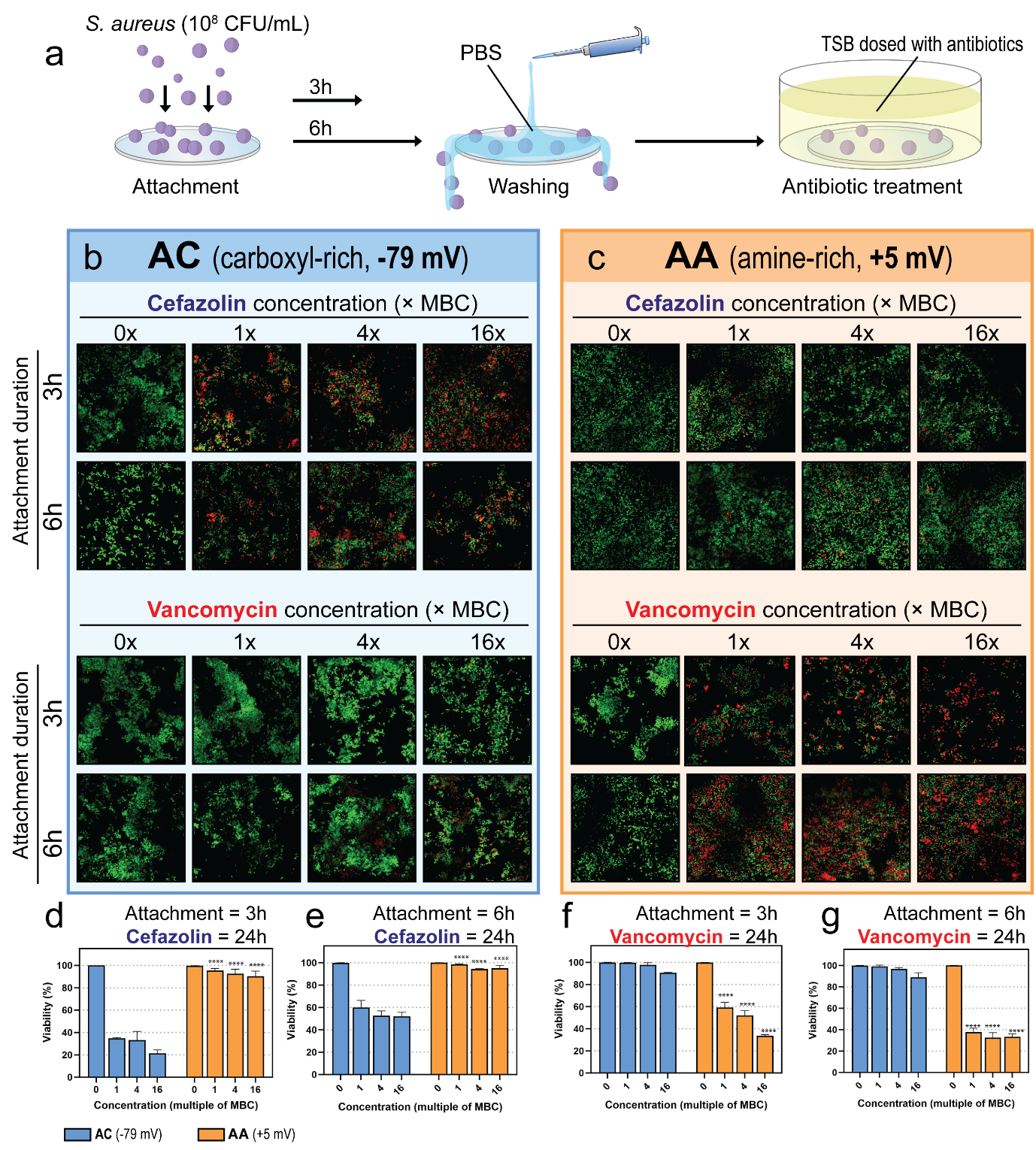


**Fig. S9**. The activity of oppositely charged antibiotics (i.e. cefazolin = -1.0 *e* and vancomycin = +0.9 *e*) against *S. aureus* attached to charged surfaces, given 3 and 6 h attachment prior to treatment. **a)** Schematic of the attachment and antibiotic treatment protocol. **b** and **c)** Fluorescence micrographs of *S. aureus* following antibiotic treatment on AC and AA coated samples, respectively. **d—g)** The quantified cell viability of *S. aureus* following different attachment times and antibiotic treatments. Scale bar represents 40 µm, **** *P* < 0.0001, *n* = 3 ± SD

**Table S3**. Proportions of live cells of *S. aureus* ATCC25923 as measured by Live/Dead staining, following **15 and 60 min attachment** to differently charged substrates and antibiotic treatment.

| **Antibiotic and concentration** | **AC** (negative charge) | | **AA** (positive charge) | |
| --- | --- | --- | --- | --- |
|  | **Pre-treatment attachment duration** | | | |
|  | 15 mins | 60 mins | 15 mins | 60mins |
| **Cefazolin** | Bacterial viability (%) | | | |
| 0 × MBC (untreated) | 100 ± 0 | 99.9 ± 0 | 99.8 ± 0.4 | 99.3 ± 0.6 |
| 1 × MBC | 14.0 ± 7.3 | 6.6 ± 3.8 | 94.3 ± 3.0 | 97.7 ± 2.3 |
| 4 × MBC | 11.9 ± 5.3 | 4.8 ± 1.3 | 81.9 ± 4.3 | 96.7 ± 1.2 |
| 16 × MBC | 6.7 ± 1.7 | 4.5 ± 2.6 | 89.0 ± 6.5 | 94.5 ± 3.1 |
| **Vancomycin** |  | | | |
| 0 × MBC (untreated) | 100 ± 0 | 99.9 ± 0.0 | 99.9 ± 0.1 | 99.3 ± 0.6 |
| 1 × MBC | 97.5 ± 0.5 | 99.9 ± 0.1 | 30.3 ± 3.2 | 43.6 ± 14.9 |
| 4 × MBC | 94.4 ± 0.7 | 94.0 ± 2.0 | 26.0 ± 1.5 | 13.1 ± 1.8 |
| 16 × MBC | 87.8 ± 3.0 | 90.6 ± 2.1 | 13.1 ± 8.2 | 7.6 ± 3.3 |

**Table S4.** Proportions of live cells of *S. aureus* ATCC25923 as measured by live/dead staining, following **3 and 6 h attachment** to differently charged substrates and treatment with antibiotics.

| Antibiotic and concentration | AC (negative charge) | | AA (positive charge) | |
| --- | --- | --- | --- | --- |
|  | Pretreatment attachment duration | | | |
|  | 3 h | 6 h | 3 h | 6 h |
| Cefazolin | Bacterial viability (%) | | | |
| 0 × MBC (untreated) | 100 | 99.9 ± 0.1 | 99.6 ± 0.2 | 100 |
| 1 × MBC | 34.7 ± 0.8 | 60.0 ± 6.4 | 95.4 ± 1.9 | 98.5 ± 0.4 |
| 4 × MBC | 33.2 ± 7.8 | 52.6 ± 4.4 | 92.6 ± 4.0 | 94.3 ± 0.4 |
| 16 × MBC | 21.3 ± 3.2 | 52.2 ± 3.5 | 90.4 ± 4.5 | 95.0 ± 2.5 |
| Vancomycin |  | | | |
| 0 × MBC (untreated) | 100 | 99.9 ± 0.1 | 99.6 ± 0.2 | 100 |
| 1 × MBC | 99.7 ± 0.2 | 99.0 ± 1.4 | 59.2 ± 4.6 | 37.6 ± 3.8 |
| 4 × MBC | 97.7 ± 2.0 | 96.8 ± 1.3 | 52.1 ± 4.2 | 32.4 ± 4.8 |
| 16 × MBC | 90.7 ± 0.5 | 89.0 ± 4.2 | 33.6 ± 0.9 | 33.2 ± 2.8 |

*Efficacy of antibiotics against methicillin-resistant S. aureus incubated on charged substrates*


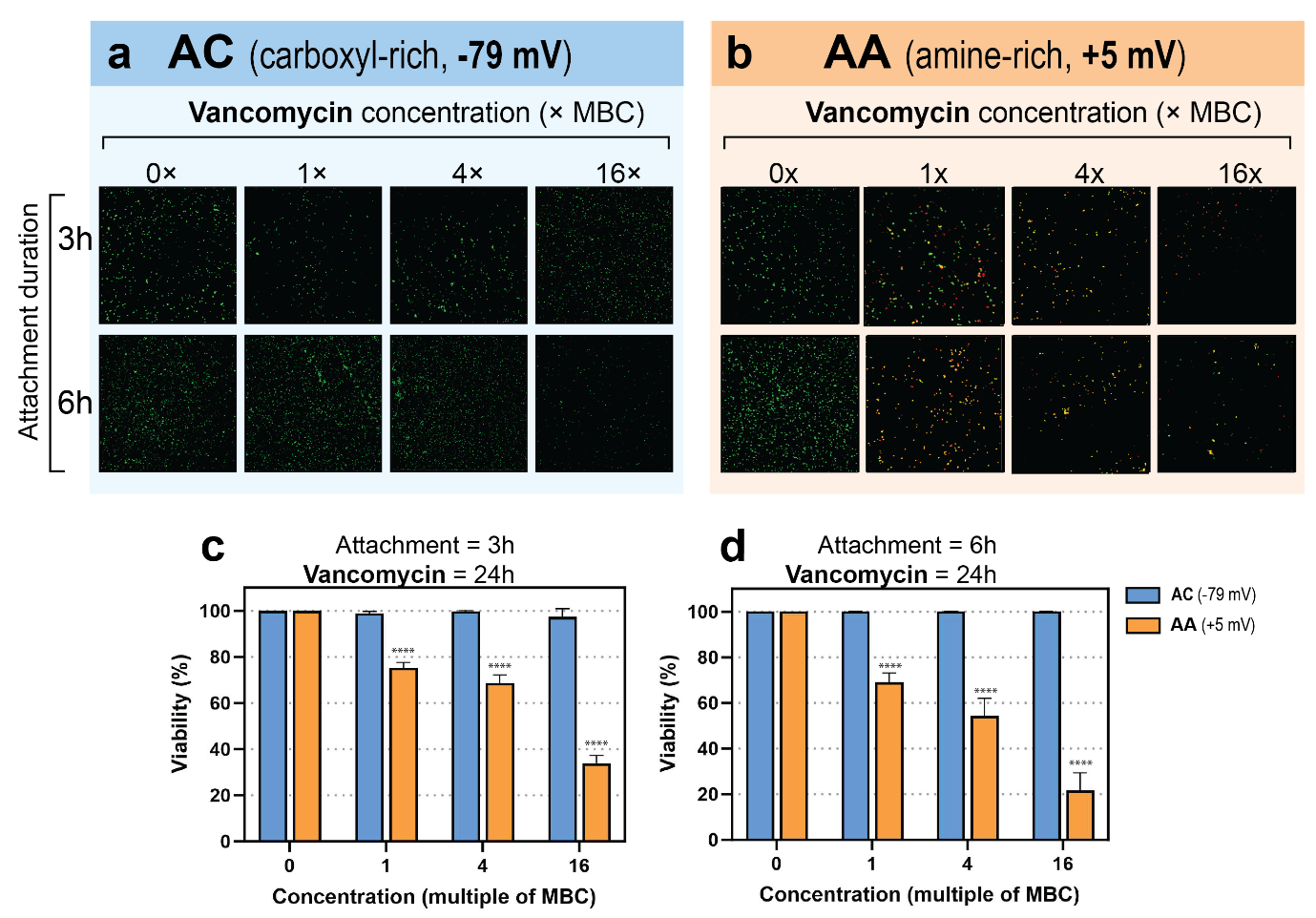


**Fig. S10.** The efficacy of vancomycin against *S. aureus* ATCC700699 (MRSA) attached to oppositely charged surfaces. **a and b)** Fluorescence micrographs of *S. aureus* attached to AA and AC and stained with Baclight Live/Dead. **c and d**) quantified viability measurements of MRSA

**Table S5.** Proportions of live cells of *S. aureus* (MRSA) as measured by live/dead staining, following **15- and 60-mins attachment** to differently charged substrates and 24h antibiotic treatment.

| **Vancomycin concentration** | **AC** (negative charge) | | **AA** (positive charge) | |
| --- | --- | --- | --- | --- |
|  | **Pre-treatment attachment duration** | | | |
|  | 15 mins | 60 mins | 15 mins | 60 mins |
| 0 × MBC | 100 ± 0 | 99.9 ± 0.0 | 99.9 ± 0.1 | 99.3 ± 0.6 |
| 1 × MBC | 97.5 ± 0.5 | 99.9 ± 0.1 | 30.3 ± 3.2 | 43.6 ± 14.9 |
| 4 × MBC | 94.4 ± 0.7 | 94.0 ± 2.0 | 26.0 ± 1.5 | 13.1 ± 1.8 |
| 16 × MBC | 87.8 ± 3.0 | 90.6 ± 2.1 | 13.0 ± 8.2 | 7.6 ± 3.3 |

**Table S6.** Proportions of live cells of *S. aureus* ATCC700699 (MRSA) as measured by live/dead staining, following **3 and 6 h attachment** to differently charged substrates and treatment with vancomycin.

| Vancomycin concentration | AC (negative charge) | | AA (positive charge) | |
| --- | --- | --- | --- | --- |
|  | Pretreatment attachment duration | | | |
|  | 3 h | 6 h | 3 h | 6 h |
| 0 × MBC | 100.0 ± 0 | 100.0 ± 0 | 100 ± 0 | 100.0 |
| 1 × MBC | 98.8 ± 0.8 | 100.0 ± 0.1 | 75.3 ± 2.4 | 69.1 ± 4.1 |
| 4 × MBC | 99.6 ± 0.5 | 99.9 ± 0.1 | 68.7 ± 3.5 | 54.3 ± 7.7 |
| 16 × MBC | 97.2 ± 3.7 | 100.0 ± 0.1 | 33.9 ± 3.4 | 21.6 ± 7.7 |

**
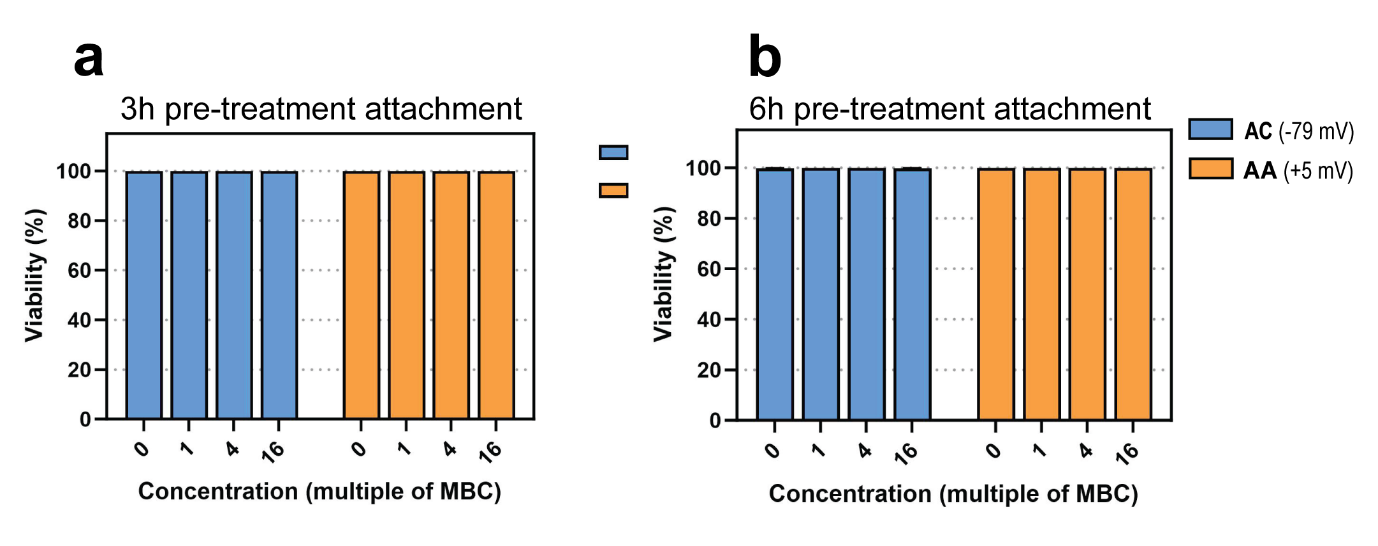
**

**Fig. S11.** The lack of activity of cefazolin against MRSA, regardless of surface type. Viability of *S. aureus* ATCC700699 after **(a)** 3 h pre-treatment attachment and exposure to cefazolin and **(b)** 6 h pre-treatment attachment.

**References**

[1] Z. Jia, M.L. O'Mara, J. Zuegg, M.A. Cooper, A.E. Mark, Vancomycin: ligand recognition, dimerization and super-complex formation, The FEBS Journal 280(5) (2013) 1294-1307.

[2] S. Watanabe, M. Tsuda, T. Terada, T. Katsura, K. Inui, Reduced renal clearance of a zwitterionic substrate cephalexin in MATE1-deficient mice, J Pharmacol Exp Ther 334(2) (2010) 651-6.

[3] E.J. Johnson, E.T. Zemanick, F.J. Accurso, B.D. Wagner, C.E. Robertson, J.K. Harris, Molecular Identification of Staphylococcus aureus in Airway Samples from Children with Cystic Fibrosis, PLOS ONE 11(1) (2016) e0147643.

[4] T. Koprivnjak, V. Mlakar, L. Swanson, B. Fournier, A. Peschel, P. Weiss Jerrold, Cation-Induced Transcriptional Regulation of the dlt Operon of Staphylococcus aureus, Journal of Bacteriology 188(10) (2006) 3622-3630.

[5] S.-J. Yang, S. Bayer Arnold, N. Mishra Nagendra, M. Meehl, N. Ledala, R. Yeaman Michael, Q. Xiong Yan, L. Cheung Ambrose, The Staphylococcus aureus Two-Component Regulatory System, GraRS, Senses and Confers Resistance to Selected Cationic Antimicrobial Peptides, Infect Immun 80(1) (2012) 74-81.

[6] CLSI, Performance Standards for Antimicrobial Susceptibility Testing. 30th ed, Clinical and Laboratory Standards Institute2020.
